# Exploring *Histoplasma* species seroprevalence and risk factors for seropositivity in The Gambia’s working equid population: Baseline analysis of the Tackling Histoplasmosis project dataset

**DOI:** 10.3389/fvets.2024.1444887

**Published:** 2024-09-19

**Authors:** Tessa Rose Cornell, Biram Laity Fye, Edrisa Nyassi, Fatou Ceesay, Mahmud Jallow, R. Frèdi Langendonk, Dan G. Wootton, Gina Pinchbeck, Claire Elizabeth Scantlebury

**Affiliations:** ^1^Institute of Infection, Veterinary, and Ecological Sciences (IVES), University of Liverpool, Liverpool, United Kingdom; ^2^Department of Livestock Services (DLS), Ministry of Agriculture, Livestock and Food Security, Abuko, Gambia; ^3^NIHR Health Protection Research Unit in Emerging and Zoonotic Diseases, University of Liverpool, Liverpool, United Kingdom

**Keywords:** *Histoplasma*, The Gambia, equine epizootic lymphangitis, equine histoplasmosis, cross-sectional study, seroprevalence, infection prevalence, working equids

## Abstract

**Introduction:**

Exposure rates to *Histoplasma* species, the causative agent of equine epizootic lymphangitis (EL), are unknown amongst working equids in The Gambia. The primary aims of this study were to estimate anti-*Histoplasma* antibody seroprevalence in the equid population in rural The Gambia and to explore risk factors for seropositivity.

**Methods:**

A nationwide cross-sectional study was conducted (February–July 2022), representing baseline measurements of a longitudinal cohort study. Horses (*n =* 463) and donkeys (*n* = 92) without EL signs were recruited in 18 study sites. Following informed owner consent, equid clinical and management data were recorded. Blood samples were collected by jugular venepuncture, and sera were subject to the IMMY Latex Agglutination *Histoplasma* test (LAT). Seropositivity risk factors were explored by multi-level, multivariable logistic regression analysis. Study site and household variance were described using a latent-variable approach. Whole blood DNA extractions were subject to nested ITS-PCR to detect *Histoplasma capsulatum* var. *farciminosum* (HCF), and agreement with LAT results was measured using Cohen’s kappa statistic.

**Results:**

Anti-*Histoplasma* antibody seroprevalence in horses and donkeys was 79.9% [95% confidence interval (CI) 76.0–83.5%] and 46.7% (95% CI 36.3–57.4%), respectively. In *horses*, two multivariable models explained the maximum amount of data variability. Model 1 demonstrated increased odds of seropositivity in mares [odds ratio (OR) = 2.90 95% CI 1.70–4.95, *p* < 0.001] and decreased odds in horses <2.5 years (OR = 0.46 95% CI 0.22–0.95, *p* = 0.04; reference: ≥4.5 years). Model 2 demonstrated increased odds in horses recruited during the rainy season (OR = 2.03 95% CI 1.08–3.84, *p* = 0.03) and those owned by farmers reporting previous EL in their equids (OR = 1.87 95% CI 1.04–3.37, *p* = 0.04). Decreased odds were measured in horses <2.5 years (OR = 0.37 95% CI 0.18–0.78, *p* = 0.01) and horses reported to transport firewood (OR = 0.45 95% CI 0.28–0.74, *p* = 0.001). On multivariable analysis of *donkeys*, decreased odds of seropositivity were demonstrated amongst donkeys owned by households which also owned horses (OR = 0.23 95% CI 0.06–0.85, *p* = 0.03). HCF infection prevalence in horses and donkeys was 22.0% (*n* = 102/463, 95% CI 18.3–26.1%) and 5.4% (*n* = 5/92, 95% CI 1.8–12.2%), respectively. No significant agreement was measured between LAT and nested ITS-PCR results (*κ* < 0.00).

**Conclusion:**

High *Histoplasma* spp. exposure was demonstrated amongst equids in The Gambia. Investigation of risk factors, including equid husbandry and management strategies, as well as geoclimatic variations, is warranted. Outcomes may inform sustainable and equitable EL control strategies in The Gambia and comparable settings worldwide.

## 1 Introduction

Despite a global working equid population of 100–112 million, with an estimated 36 million equids residing in the 38 least developed countries, equid population data are lacking or inaccurate in many settings (1). These evidence gaps are partly driven by the under-recognition of the economic impact of working equids and their low societal status compared to ruminant livestock species. Working equids make significant contributions to livelihoods in low- and middle-income countries (LMICs), through frontline roles in agricultural, transportation, and construction sectors (2, 3). The chronic progressive nature of equine EL, associated with *Histoplasma capsulatum* variety (var) *farciminosum* (HCF), reduces the working capacity of equids, with significant socioeconomic implications (4–8). Across West Africa, published evidence of EL in horses comprises historic or limited case reports in Senegal (9) and Nigeria (10). In The Gambia, anecdotal evidence comprises sporadic case and outbreak reports of EL, with an increased frequency observed during the rainy season [Department of Livestock Services (DLS), Ministry of Agriculture, The Gambia, personal communication]. Furthermore, in The Gambia, molecular detection of HCF from equid clinical samples has been confirmed (11). Limited national EL surveillance, reporting, and control efforts are compounded by poor EL treatment access and affordability, as well as non-mandatory disease notification. Although injectable sodium iodide preparation has been sourced from Senegal periodically for cutaneous EL treatment (DLS, personal communication) as described in the literature (12), this regimen presents limitations in relation to access, affordability, treatment duration, and ease of administration by equid owners. International EL control and prevention guidelines describe culling infected equids and strict adherence to hygiene practices (13, 14). However, these may be prohibited in LMIC contexts, including in The Gambia, due to the associated socioeconomic implications and lack of compensation for equid loss. In Ethiopia, the effectiveness of alternative interventions on reducing EL prevalence has been demonstrated, including wound management strategies, harness assessment, and educational interventions for equid owners (15).

A formal examination of risk factors for *Histoplasma* species exposure to support context-specific EL control strategies has not been carried out in The Gambia. Furthermore, the recognition of *Histoplasma* spp. as “high priority” human pathogenic fungi with established environmental reservoirs (16–19) and the visibility of human-animal-environment interfaces across rural The Gambia, justify robust investigations of the epidemiology of *Histoplasma* spp. across mammalian host populations using a One Health approach.

This cross-sectional study analysed the baseline dataset of a longitudinal cohort study that explored the *Histoplasma* spp. burden in the working equid population of The Gambia.

The objectives of this study were to: (i) estimate anti-*Histoplasma* antibody seroprevalence using the IMMY LAT; (ii) explore equid demographic, clinical, and management risk factors for seropositivity by multi-level multivariable logistic regression analysis; (iii) estimate HCF infection prevalence using a nested ITS-PCR technique; and (iv) compare LAT results with nested ITS-PCR results.

## 2 Materials and methods

### 2.1 Study design and recruitment procedures

**Figure 1:**
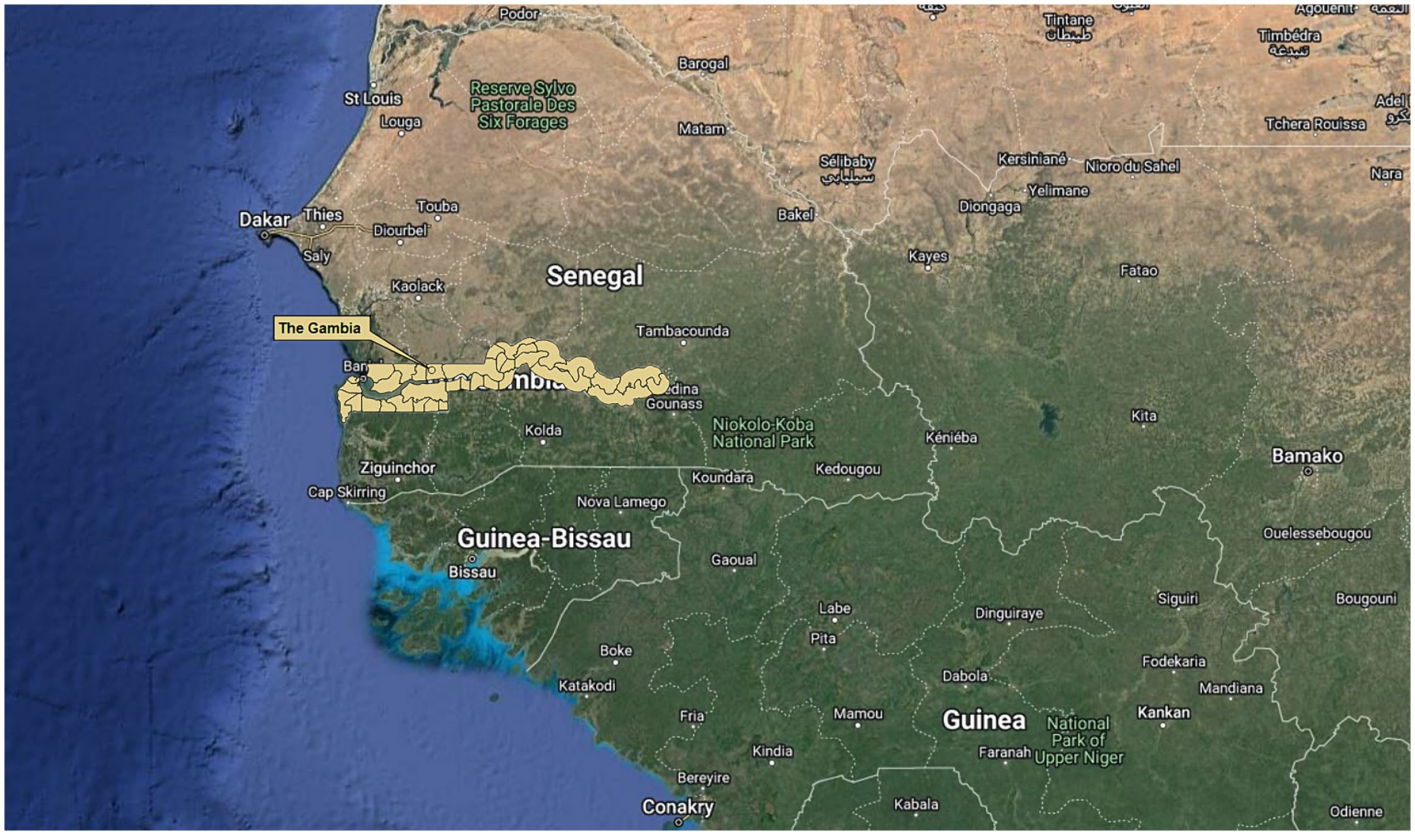
Map of The Gambia, West Africa. Boundary lines represent district borders. Geodata were downloaded from Google Maps Satellite Imagery and DIVA-GIS (https://www.diva-gis.org/gdata). DIVA-GIS software is a free and open-access source (https://www.diva-gis.org/docs/DIVA-GIS5_manual.pdf). Maps were generated using QGIS 3.12.

**Figure 2:**
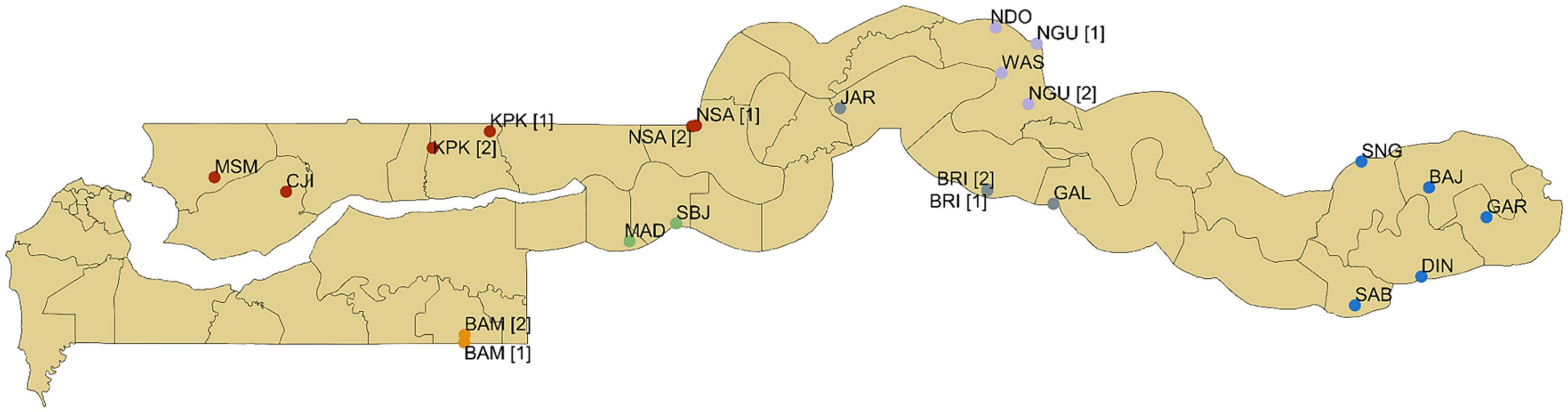
Study site locations in The Gambia. Colour of study site location points relates to administrative regions (from west to east): red = North Bank Region; orange = West Coast Region; green = Lower River Region; grey = Central River Region South; purple = Central River Region North; blue = Upper River Region. In five study sites (codes BAM, KPK, NSA, BRI, and NGU), equid recruitment activities were divided between two locations (labelled [1] and [2]) to ensure that the target sample size was met. Coordinates were captured using a handheld Garmin eTrex 10 GPS unit. Geodata were downloaded from Google Maps Satellite Imagery and DIVA-GIS (https://www.diva-gis.org/gdata). DIVA-GIS software is a free and open-access source (https://www.diva-gis.org/docs/DIVA-GIS5_manual.pdf). The map was generated using QGIS 3.12.

Data represent baseline measurements of a nationwide prospective 16-month longitudinal cohort study examining the burden of *Histoplasma* in the equid population. Baseline data were collected from February to July 2022, in 18 study sites across six administrative regions of The Gambia (Figures 1, 2). Study sites demonstrated high equid populations compared to other settlements and relative to the human population size of selected sites, based on 2016 Livestock Census data (20) and regional knowledge (DLS, personal communication). Sites encompassed settlements with and without a *Lumo* (weekly market), and were located at variable distances from the Senegalese border (Figure 2).

As previous studies reporting the incidence of equine *Histoplasma* exposure in West Africa are lacking, sample size calculations were based on limited longitudinal data from another context (pertaining to the occurrence and distribution of EL in Ethiopia) (21). Fourteen percent of equids were estimated to develop HCF infection (21), with an assumed 95% confidence level and 80% power to detect ORs of at least 2.5 for exposures of ≥20% (22). Accounting for an anticipated loss-to-follow-up of approximately 10%, the target sample size was 540 equids (30 equids per study site).

All equid owners ≥18 years old with a permanent address in The Gambia, who could provide voluntary informed consent, were eligible to participate and enrol equids in the study. Study information was written in English and translated verbally into the languages spoken by owners (Mandinka, Fula, Serahule, Wolof, or Jola) by the research team or livestock assistants (LAs; animal health paraprofessionals, DLS). Consenting participants signed or marked the consent form, with an impartial witness of consent present if a participant was unable to read or write. All equids at study sites on pre-scheduled recruitment days and without clinical signs of EL (to explore EL incidence as part of the wider longitudinal cohort study), were eligible to participate. The sampling approach was not random, as all presenting animals at each study site on the allocated recruitment day were recruited (until the target sample size was met). Furthermore, it was noted that owners preferentially presented horses over donkeys if both species were owned. One equid per owner was recruited, with the exception of study sites with lower community attendance in order to achieve the target sample size. For each animal recruited to the study, a microchip was implanted aseptically under local anaesthesia, and a unique three-digit identifier was allocated for anonymization and data collation purposes.

### 2.2 Clinical data collection and blood sampling

A clinical examination was performed by a veterinarian (TRC) and an animal health paraprofessional (EN). Clinical data described signalment, vital parameters, body condition (0–5 scale), and ocular, respiratory, gastrointestinal, musculoskeletal and dermatological health. Age was estimated based on incisor eruption times (23).

Clinical data were recorded offline on the KoboCollect application using two digital devices and uploaded to the KoboToolbox server when the network was available. Exported datasets were stored in password-protected Microsoft Excel files.

Venous blood samples were collected from each animal via jugular venepuncture into 6 mL plain and EDTA tubes labelled with the allocated unique identifier. Samples were placed in a temperature-controlled electric cool box powered by an in-car socket (Halfords 40 L Electric Coolbox) for 1–4 h post-collection, before temporary storage at 4°C and initial processing at DLS Regional Directorates, and subsequent transportation to the project laboratory.

### 2.3 Equid owner questionnaire

A questionnaire was delivered to equid owners by three research team members, addressing equid management and clinical history. Variables encompassed plausible risk factors for *Histoplasma* spp. exposure based on current literature and related to the following areas: equid use and work status, equid restraint and housing, equid water and diet management, use of harness or cart, healthcare (encompassing hoof care and endoparasite management), and history of EL. Selected variables were also informed by piloting exercises in which the research team reviewed and trialled the questionnaire together to ensure questions were context-specific and unambiguous. GIS data were captured using a handheld Garmin eTrex 10 GPS unit. Data were recorded on the KoboCollect application as per clinical data.

### 2.4 Blood sample measurements

Packed cell volume (PCV) and total protein (TP) by refractometry were measured <24 h post-sample collection at DLS Regional Directorates. Differential cell counts (DCCs) were performed by two research team members by direct microscopy of Giemsa-stained thin blood smears. Proportions of neutrophils, lymphocytes, basophils, eosinophils, and monocytes were calculated as percentages of 200 cells counted per sample. Automated diagnostic analyzers were not available, and direct microscopy represented a practical and cost-effective method of DCC measurement.

### 2.5 Anti-*Histoplasma* antibody detection

Serum from plain tubes was spun at 2500 rpm for 15 min <24 h post-collection, and stored at 4°C prior to serological testing. Serum aliquots were heat-treated (30 min at 56°C) and subject to one IMMY LAT (Norman, OK, United States) per animal, as per the manufacturer guidelines (24). Test results were assigned using a graduated scale of reaction strengths (negative to four plus) (24). Samples assigned two plus or greater reaction strength were recorded as evidence of anti-*Histoplasma* antibody presence.

### 2.6 *Histoplasma capsulatum* var. *farciminosum* infection detection

DNA extractions were performed on the buffy coat component of whole blood samples using the Qiagen DNeasy Blood and Tissue kit (Germantown, MD, United States) (25). DNA concentration (ng/μL) and purity (260/280 ratio) of DNA extractions were analysed using a NanoDrop spectrophotometer. Aliquots of 50 ng/μL DNA were prepared using nuclease-free water and stored at −20°C.

DNA extractions were subject to a nested PCR technique using two primer sets (P3/2R8 and F5/2R5) targeting the internal transcribed spacer (ITS) 1–5.8S-ITS2 region, as described by Scantlebury et al. (26). The total reaction mixture (25 μL reaction volume) comprised: 1 μL (50 ng/μL) of template DNA, 12 μL of BioMix red, 2 μL of forward primer (P3 or F5), 2 μL of reverse primer (2R8 or 2R5), and 8 μL sterile Milli-Q water. Primers (Eurofins Genomics) were rehydrated using nuclease-free water as per manufacturer guidelines, and aliquots were prepared at 10 pmol concentration. Positive and negative controls comprised a *H. capsulatum* var. *farciminosum* reference strain and sterile Milli-Q water, respectively. Thermocycler conditions were applied, as described by Scantlebury et al. (26), to conduct two rounds of PCR.

After the second round of PCR, the expected product was 514 base pairs (bp) and was visualised using the gel electrophoresis technique. A 1% (wt/vol) agarose gel was prepared and stained with Midori green (8 μL per 100 mL agarose). The gel was run with a 1 kb hyper-ladder (Bioline) at 70–100 V for 20–30 min and visualised in a lightbox under ultraviolet light or on a blue light transilluminator.

Each DNA extraction was subject to a repeat PCR. If concordance was not met between the first and second PCR results or if any result was ambiguous (faint band on gel visualisation), the DNA extraction was subject to a third PCR. PCR products demonstrating a band of 514 bp on gel electrophoresis for 2/2 or 2/3 nested PCRs were recorded as positive and presumptive evidence of HCF infection. PCR products demonstrating no or faint bands for 2/2 or 2/3 nested PCRs were recorded as negative. Second-round PCR products were stored at −20°C. PCRs were conducted at the University of Liverpool as no thermocyclers were available at project sites in The Gambia at the time of this study.

### 2.7 Statistical analysis

#### 2.7.1 Study population baseline characteristics

Baseline characteristics of the study population were explored using descriptive statistics. Equid age and sex distributions of study and national populations were compared using the 2016 Livestock Census data (20). Horse and donkey populations were examined separately to compare species-specific demographic, clinical, and management profiles.

#### 2.7.2 Seroprevalence estimation

Seroprevalence of anti-*Histoplasma* antibody and 95% CIs were estimated using the Epitools interface and Clopper-Pearson (exact) test (27). Seroprevalence at household, study site, and administrative region levels was estimated by identifying at least one equid demonstrating *Histoplasma* seropositivity at each geographic level.

Apparent seroprevalence adjusted for clustering at household and study site levels was calculated using the constant parameter estimate derived from the random intercept-only multi-level models from MLwiN 3.05 software.

#### 2.7.3 Univariable analysis

Risk factors for *Histoplasma* spp. seropositivity were explored for horse and donkey study populations separately, based on context-specific clinical and management variations by equid species and current literature which demonstrates higher EL prevalence in horses compared to donkeys (28).

Univariable associations were examined between *Histoplasma* spp. serostatus, and demographic, seasonal, clinical, and management variables. Univariable logistic regression, with *Histoplasma* spp. serostatus as the binary outcome, was applied. ORs with 95% CIs and associated *p*-values were presented.

Temporal data regarding timing of *Histoplasma* spp. exposure were not available; thus, to avoid inappropriate inferences, associations with seropositivity were not explored for variables that could only be considered indicative of clinical status at the time of sampling (heart rate, respiratory rate, temperature, gastrointestinal sounds on auscultation, and digital pulses).

One serum sample was not available for serological testing; thus, this horse was excluded from subsequent regression analysis.

#### 2.7.4 Multi-level multivariable logistic regression analysis

Variables with a *p* < 0.20 on univariable analysis were tested in multivariable logistic regression models with *Histoplasma* spp. serostatus as the binary outcome. Models were built using a manual backwards-stepwise approach (29). This analysis represents an exploratory hypothesis-generating exercise; thus, all variables with a *p* < 0.20 on univariable analysis were analysed, irrespective of whether retention was supported by established risk factors for *Histoplasma* spp. exposure in the literature.

Phi and Spearman rank coefficients, as well as *χ*^2^ test, were applied to explore inter-correlations between variables tested in multivariable models. Correlation coefficient values of >0.7 with associated *p* < 0.05 were interpreted as evidence of inter-correlations. Subsequent *p*-value comparison on univariable analysis supported subsequent variable retention or exclusion. Influential points with a delta-beta of <−0.1 or > 0.1 were excluded, and the model output was re-examined.

Random effects were added to explore the clustering of seropositivity at household and study site levels using second-order predictive quasi-likelihood (PQL) approximation method. Regression coefficients estimate *p*-values, and *z*-ratios were compared between single and multi-level models. The proportion of variance attributable to individual levels was calculated using a latent-variable approach (30).

Data analyses were performed using IBM SPSS Statistics 27 and MLwiN 3.05 software.

#### 2.7.5 Infection prevalence estimation

Apparent and true HCF infection prevalence using nested ITS-PCR technique (26) were determined, using the Epitools interface and Clopper-Pearson (exact) test to calculate 95% CIs (27). Apparent infection prevalence adjusted for clustering at household and study site levels was calculated using the constant parameter estimate derived from the random intercept-only multi-level models from MLwiN 3.05 software.

Agreement between LAT and nested ITS-PCR results in horse and donkey study populations was explored using Cohen’s kappa statistic (*κ*) and interpreted using published guidelines (31).

## 3 Results

### 3.1 Study population baseline characteristics

The study population comprised 463 horses (83.4%) and 92 donkeys (16.6%) without EL signs. Excluding one study site, the majority of animals recruited in all sites were horses (Supplementary Table S1). Donkeys were recruited in 10 sites across 6 regions. Owners preferentially presented horses (if horses *and* donkeys were owned), reflecting the increased socioeconomic value of horses in The Gambia (study participants, personal communication).

The study population originated from 439 households, of which 19.8% (*n* = 87/439) owned >1 study equid. In the event of low community participation, >1 equid was recruited per owner to achieve the target study site sample size. The majority of owners reported owning other horses (*n* = 420/463, 90.7%) or donkeys (*n* = 70/92, 76.1%). Excluding the equid owner, the median number of human occupants per compound was 16.0 (IQR = 11.0–26.0, range = 0–114). A *compound* refers to the physical area where one or more households (group(s) of persons living together and sharing from the same food bowl) reside.

Demographic and clinical characteristics are presented in Supplementary Tables S2–S4. Compared to the 2016 Livestock Census, the study population demonstrated a higher percentage of male horses (*n* = 259/463, 55.9%; compared to 44.9% in the census) but percentages of male donkeys were comparable (20). Male study horses comprised stallions (*n* = 258/259, 99.6%) and only one gelding. The age distribution demonstrated a negative skew, with the majority of horses (*n* = 373/463, 80.6%) and donkeys (*n* = 79/92, 85.9%) aged ≥4.5 and ≥ 5.5 years based on incisor eruption times, respectively (Supplementary Table S2).

A significant association was measured between horse sex and body condition score (BCS; *χ^2^*-associated *p* < 0.001), with a higher proportion of mares demonstrating BCS 0–1 or 2/5, and a higher proportion of stallions demonstrating BCS 3. Owners reported that 13.7% (*n* = 28/204) of mares and 20.4% (*n* = 10/49) of jennies were in foal.

Previous and resolved cutaneous EL occurrence in any owned equid was reported by a higher proportion of horse owners (*n* = 138/463, 29.8%; donkey owners: *n* = 14/92, 15.2%; *χ^2^*-associated *p* < 0.05). Owners of four study horses and one donkey (*n* = 5/555, 0.9%) reported ongoing EL cases in other owned equids based on the observation of EL-suggestive skin lesions (Supplementary Table S2). Non-EL wounds were observed in a higher proportion of donkeys (*n* = 31/92, 33.7%; horses: *n* = 103/463, 22.2%; *χ^2^*-associated *p* < 0.05; Supplementary Table S4).

Equid management variables are presented in Supplementary Table S5. The two most frequently reported uses of equids were ploughing (horses: 93.7%, *n =* 434/463; donkeys: 91.3%, *n* = 84/92) and transportation of firewood (horses: 47.1%, *n* = 218/463; donkeys: 81.5%, *n* = 75/92). During the dry season, 75.0% (*n* = 69/92) of donkeys were engaged in work, whereas 54.0% (*n* = 250/463) of horses were *not* working. During the rainy season, a comparable proportion of horses (*n* = 423/463, 91.4%) and donkeys (*n* = 87/92, 94.6%) were working (Supplementary Table S5).

### 3.2 Seroprevalence

Anti-*Histoplasma* antibody seroprevalence of 79.9% (*n* = 370/463, 95% CI 76.0–83.5%) and 46.7% (*n* = 43/92, 95% CI 36.3–57.4%) were measured in horses and donkeys, respectively. The majority of horse and donkey sera displayed a reaction strength of 2+ (*n* = 196/463, 42.3%) and 1+ (*n* = 47/92, 51.1%), respectively (Table 1). Table 2 presents apparent seroprevalence values adjusted for clustering at household and study site levels.

**Table 1:** Frequency distribution of IMMY latex agglutination *Histoplasma* test results for horses (*N* = 463) and donkeys (*N* = 92), categorised by reaction strength and result interpretation.

	Reaction strength	Description (IMMY, 24)	Horses, *n* (%), total *N* = 463	Donkeys, *n* (%), total *N* = 92
Negative LAT	-	A homogeneous suspension of particles with no visible clumping	0 (0.0)	2 (2.2)
	1+	Fine granulation against a milky background	92 (19.9)	47 (51.1)
Positive LAT	2+	Small but definite clumps against a slightly cloudy background	196 (42.3)	28 (30.4)
	3+	Large and small clumps against a clear background	147 (31.7)	13 (14.1)
	4+	Large clumps against a very clear background	27 (5.8)	2 (2.2)
Missing serum			1 (0.2)	–
LAT result interpretation	Negative	Reaction strength – or 1+	92 (19.9)	49 (53.3)
	Positive	Reaction strength 2+ to 4+	370 (79.9) [95% CI 76.0–83.5]	43 (46.7) [95% CI 36.3–57.4]
	Missing		1 (0.2)	–

**Table 2:** Apparent prevalence of anti-*Histoplasma* antibody using latex agglutination test (LAT) and infection prevalence using nested ITS-PCR, amongst horses (*N* = 463) and donkeys (*N* = 92), unadjusted and adjusted for clustering at household and study site levels.

		Horses, *N* = 463		Donkeys, *N* = 92	
		Prevalence, %	95% CI	Prevalence, %	95% CI
Apparent seroprevalence using LAT	Unadjusted	79.9	76.0–83.5	46.7	36.3–57.4
	Adjusted	80.7	75.5–85.1	47.1	34.2–60.5
Apparent infection prevalence using nested ITS-PCR	Unadjusted	22.0	18.3–26.1	5.4	1.8–12.2
	Adjusted	14.6	8.3–24.4	5.5	2.3–12.5

Prevalence values and 95% confidence intervals (CIs) were calculated using Epitools Interface and Clopper–Pearson (exact) test and MLwiN 3.05 software.

Of households represented by study horses, 82.0% (*n* = 306/373) owned at least one seropositive horse, compared to households represented by study donkeys of which 47.0% (*n* = 39/83) owned at least one seropositive donkey. At least one horse demonstrated seropositivity across all study sites, and at least one donkey demonstrated seropositivity in 80.0% (*n* = 8/10) of study sites across five regions (Supplementary Table S1).

### 3.3 Univariable logistic regression analysis

Univariable logistic regression analyses are presented in Supplementary Tables S6–S9. Amongst horses, statistically significant associations were measured between *Histoplasma* spp. seropositivity and the following demographic variables: sex (female: OR = 2.66 95% CI 1.60–4.41, *p* < 0.001), age (<2.5 years: OR = 0.50 95% CI 0.25–0.99, *p* = 0.045, reference category: ≥4.5 years) and foaling history [previously in foal (owner-reported): OR = 4.90 95% CI 1.02–23.53, *p* = 0.047; Supplementary Table S6]. On univariable analysis, no statistically significant associations were measured between haematological or biochemical parameters and *Histoplasma* spp. seropositivity (Supplementary Table S7). Horses recruited in the Central River Region/North demonstrated significantly lower odds of *Histoplasma* spp. seropositivity [OR = 0.47 95% CI 0.25–0.90, *p* = 0.02; reference category: Upper River Region (URR) recruitment; Supplementary Table S8]. This variable lost significance following the inclusion of study site as a random effect on multi-level analysis. Statistically significant associations were measured between *Histoplasma* spp. seropositivity and management variables pertaining to the following: methods of restraint, use of the horse in multiple transport or construction roles, travel to Senegal to visit *Lumos* or for other purposes, and work status during the dry season (Supplementary Table S9).

Amongst donkeys, no statistically significant associations were measured between *Histoplasma* spp. seropositivity and demographic variables (Supplementary Table S6). Statistically significant associations were measured between seropositivity, and both PCV (OR = 1.09 95% CI 1.01–1.19, *p* = 0.04), and recruitment in Central River Region/ South (OR = 0.13 95% CI 0.03–0.58, *p* = 0.01; reference category: recruitment in URR; Supplementary Tables S7, S8). As demonstrated by univariable analysis in horses, associations between seropositivity and management variables pertaining to the use of the donkey in multiple transport or construction roles were statistically significant (Supplementary Table S9).

### 3.4 Multi-level multivariable logistic regression analysis

#### 3.4.1 Horse study population

Two three-level models were proposed, with both including random effects to allow for clustering by household and study site. During model building using a backward-stepwise approach, the significance of retained variables based on associated *p*-values supported variable retention or exclusion.

A correlation was measured between sex and previous EL in any other owned equid. As the variable describing previous EL refers to *any* owned equid and is not specific to the study animal, both variables were considered to be independently theoretically plausible as risk factors for seropositivity and were retained in alternative models. Furthermore, inter-correlations were demonstrated between sex and equid management variables (tested one-by-one and in combination in the model), including variables describing equid use for transportation or construction, work engagement during the dry season, and method of restraint during the day when resting (loose vs. tethered).

Model 1 contained two statistically significant main effects: female horses (OR = 2.90 95% CI 1.70–4.95, *p* < 0.001) and age category <2.5 years on dental examination (OR = 0.46 95% CI 0.22–0.95, *p* = 0.04; reference category: ≥4.5 years; Table 3). No data points were influential on the outcome of model 1 based on delta-beta analysis.

**Table 3:** Model 1: Multi-level multivariable logistic regression, examining variable associations with *Histoplasma* spp. seropositivity based on LAT results, amongst horses (*N* = 463) in The Gambia.

Variable	Frequency, *n* (%), total *N* = 463	*Histoplasma* seropositive, *n* (%), *N* = 370^a^	*Histoplasma* seronegative, *n* (%), *N* = 92^a^	Odds Ratio (95% CIs)	*p-*value
Main effects					
*Sex*					
Male (ref)	259 (55.9)	191 (73.7)	68 (26.3)	1.00	
Female	204 (44.1)	179 (88.2)	24 (11.8)	2.90 (1.70–4.95)	<0.001*
*Age, years*					
<2.5	44 (9.5)	30 (68.2)	14 (31.8)	0.46 (0.22–0.95)	0.037*
2.5–4.5	46 (9.9)	38 (82.6)	8 (17.4)	1.09 (0.47–2.56)	0.837
≥4.5 (ref)	373 (80.6)	302 (81.2)	70 (18.8)	1.00	
Random effects					
*Household*					
Variance (standard error)	0.00 (0.00)				
*Study site*					
Variance (standard error)	0.24 (0.17)				

Household and study sites are included as random effects. Descriptive statistics, odds ratios (ORs), 95% confidence intervals (CIs), and *p*-values were calculated using MLwiN 3.05 software. ^*^p < 0.05 (statistically significant). ^a^*n* = 1 horse excluded based on missing serum sample (no LAT result).

Clustering by study site was demonstrated, indicating that 6.8% of the variance in seropositivity was due to the study site using the latent-variable approach. No evidence for clustering at the household level was demonstrated. Season and region were included as the main effects, but lost significance following the inclusion of the study site as a random effect.

To explain the maximum amount of variability in the data, model 2 was proposed, which excluded sex and encompassed variables pertaining to both previous EL and the use of equid for firewood transport (Table 4). Model 2 retained the main effect, age (OR = 0.37 95% CI 0.18–0.78, *p* = 0.009; reference category: ≥4.5 years). Following the exclusion of sex, the model retained the following statistically significant and theoretically plausible main effects: previous EL in any owned equid (OR = 1.87 95% CI 1.04–3.37, *p* = 0.04), recruitment during the rainy season (OR = 2.03 95% CI 1.08–3.84, *p* = 0.03), and use of equid to transport firewood (OR = 0.45 95% CI 0.28–0.74, *p* = 0.001). No data points were influential on the model 2 outcome based on delta-beta analysis.

**Table 4:** Model 2: multi-level multivariable logistic regression examining variable associations with *Histoplasma* spp. seropositivity based on LAT results, amongst horses (*n* = 463) in The Gambia.

Variable	Frequency, *n* (%), total *N* = 463	*Histoplasma* seropositive, *n* (%), *N* = 370 ^a^	*Histoplasma* seronegative, *n* (%), *N* = 92 ^a^	Odds ratio (95% CI)	*p-*value
Main effects					
*Age, years*					
<2.5	44 (9.5)	30 (68.2)	14 (31.8)	0.37 (0.18–0.78)	0.009*
2.5–4.5	46 (9.9)	38 (82.6)	8 (17.4)	0.98 (0.42–2.29)	0.954
≥4.5 (ref)	373 (80.6)	302 (81.2)	70 (18.8)	1.00	
*Previous EL in any owned equid*					
No (ref)	324 (70.0)	251 (77.7)	72 (22.3)	1.00	
Yes	138 (29.8)	118 (85.5)	20 (14.5)	1.87 (1.04–3.37)	0.037*
NR/ND	1 (0.2)	1 (100.0)	0 (0.0)	-	-
*Season at recruitment*					
Dry (November–May) (ref)	325 (70.2)	252 (77.8)	72 (22.2)	1.00	
Rainy (June–October)	138 (29.8)	118 (85.5)	20 (14.5)	2.03 (1.08–3.84)	0.028*
*Firewood transport*					
No (ref)	245 (52.9)	209 (85.7)	35 (14.3)	1.00	
Yes	218 (47.1)	161 (73.9)	57 (26.1)	0.45 (0.28–0.74)	0.001*
Random effects					
*Household*					
Variance (Standard Error)	0.00 (0.00)				
*Study site*					
Variance (Standard Error)	0.11 (0.13)				

Household and study sites are included as random effects. Descriptive statistics, odds ratios (ORs), 95% confidence intervals (CIs), and *p*-values were calculated using MLwiN 3.05 software. *p < 0·05 (statistically significant); EL, epizootic lymphangitis; NR/ND, no response/no data. ^a^*n* = 1 horse excluded based on the missing serum sample (no LAT result).

Model 2 demonstrated low-level study site clustering, indicating that 3.2% of the variance in seropositivity was attributable to the study site (Table 4).

#### 3.4.2 Donkey study population

A multi-level model was proposed, including random effects to estimate and allow for clustering in seropositivity attributable to household and study site. The model contained one statistically significant main effect: horse(s) owned by household (OR = 0.23 95% CI 0.06–0.85, *p* = 0.03; Supplementary Table S10). No single data point was influential on model outcome based on delta-beta analysis.

Clustering by study site was demonstrated, reflecting 16.5% of seropositivity variance due to the study site. No evidence for clustering at the household level was demonstrated.

### 3.5 Infection prevalence

Apparent asymptomatic infection prevalence of 22.0% (*n* = 102/463, 95% CI 18.3–26.1%) and 5.4% (*n* = 5/92, 95% CI 1.8–12.2%) was measured in horses and donkeys, respectively, using a nested ITS-PCR (Table 1).

No significant agreement was measured between results from the LAT and the nested ITS-PCR between horses (*κ* = −0.01, *p* = 0.60) or donkeys (κ = −0.06, *p* = 0.22; Table 5).

**Table 5:** Comparison of anti-*Histoplasma* antibody detection using latex agglutination test (LAT) and *Histoplasma capsulatum* var. *farciminosum* (HCF) infection status using nested ITS-PCR, amongst horses (*n* = 461/463) and donkeys (*N* = 92).

		HCF infection status using nested ITS-PCR, *n*					
		Horses (*n* = 461/463)			Donkeys (*N* = 92)		
		Infection negative, *n*	Infection positive, *n*	Total, *n*	Infection negative, *n*	Infection positive, *n*	Total, *n*
Anti-*Histoplasma* antibody using LAT, *n*	Seronegative	69	22	91	45	4	49
	Seropositive	290	80	370	42	1	43
	Total, *n*	359	102	461 ^a^	87	5	92

^a^ *n* = 1 horse excluded based on missing serum sample (no LAT result) with negative PCR result and *n* = 1 horse excluded based on missing DNA extraction (no PCR result) with negative LAT result.

## 4 Discussion

### 4.1 Main outcomes

Novel data are provided on anti-*Histoplasma* antibody seroprevalence in working equid populations in The Gambia, and plausible demographic, clinical, management, and seasonal risk factors for exposure are explored. Evidence of EL in The Gambia is sparse (11) (DLS, personal communication). Robust evidence of the geographic distribution of disease in The Gambia was previously non-existent due to limitations in active EL surveillance and non-mandatory disease reporting systems. To date, the World Organisation for Animal Health (WOAH) has presented country-level data on EL occurrence up to 2005 only, at which time, across the African continent, disease reports were limited to Senegal, Ethiopia, and South Africa (32). In contrast, literature on human histoplasmosis describes the widespread distribution of *Histoplasma* spp. associated with tropical and subtropical regions, in addition to increasing detection in areas previously considered non-endemic (33, 34). Across the African continent, the inclusion of EL within national disease surveillance programmes is warranted to accurately record EL geographic distribution and burden amongst equid populations. Furthermore, the impact of EL in LMICs is suspected to be compounded by a reliance on working equids to support livelihoods and a relative lack of resources to support animal health infrastructures.

Horse and donkey study populations demonstrated anti-*Histoplasma* antibody seroprevalence of 79.9 and 46.7%, respectively, indicative of high *Histoplasma* spp. exposure. The wide geographic distribution of seropositive animals provides evidence to support the endemic status of The Gambia with respect to *Histoplasma* spp. Epidemiological studies of *Histoplasma* spp. taking a One Health approach are warranted in this setting, including systematic sampling at human–animal–environment interfaces to investigate environmental reservoirs maintaining *Histoplasma* spp. mycelia in this setting. Furthermore, the public health implications of identified reservoirs and *Histoplasma* spp. transmission dynamics in a community setting may be explored by phylogenetic characterisation of isolates from human, animal and environmental origins. Across study sites where >1 donkey were also recruited (*n* = 7/18 sites), overall seroprevalence in horses (*n* = 116/140, 82.9%) was not significantly different from seroprevalence across sites where *only* horses were recruited (*n* = 254/322, 78.9%). Furthermore, overall HCF infection prevalence in horses were comparable across study sites where 0 or 1 donkey (20.7%, *n* = 69/333) and > 1 donkey (25.6%, *n* = 33/129) were recruited. Based on IgM seroconversion (targeted by the LAT), study animals demonstrating seropositivity mount an IgM response approximately 2–6 weeks post-exposure (35, 36), and before the IgM response diminishes (up to 3 months post-exposure) (35). Immunological studies exploring humoral or cell-mediated immune responses to HCF in EL cases (37) or estimating *Histoplasma* spp. seroprevalence are limited to non-existent. To date, cross-sectional studies have primarily examined infection prevalence amongst clinical cases of EL in Ethiopia, of which the majority of cases are described as the cutaneous form (28, 38–40).

The detection of HCF using a nested ITS-PCR provided definitive evidence of current infection status, compared to the LAT, which confirms exposure only. With the relative paucity of data describing asymptomatic HCF infection in equids (41), this baseline study presented serostatus as the main outcome of risk factor analysis. Thus, these data contribute novel information about the detection of fungemia in asymptomatic equids. Based on established IgM seroconversion times, study animals demonstrating seropositivity using the LAT *and* positive infection status on nested ITS-PCR, represent equids that have mounted an IgM response approximately 2–6 weeks post-exposure (35, 36) *and* developed fungemia. Amongst these apparently healthy animals, this subset (positive on LAT and PCR) represents the best evidence to date for asymptomatic carriage in equids. In humans, *Histoplasma* spp. infection is reported to be typically self-limiting (42) and evidence exists of transient fungemia in cases of acute pulmonary human histoplasmosis (43). Thus, equids may remain seropositive during the period when the IgM response is detectable, but infection negative following the resolution of fungemia, which could explain our findings reporting no overall agreement between LAT and PCR results. Further exploration of risk factors for *Histoplasma* spp. infection in asymptomatic animals and of the demographic, clinical, or environmental predictors of clinical outcome through longitudinal analysis are warranted.

Pertaining to risk factors for *Histoplasma* spp. seropositivity in horses, two multi-level multivariable models were proposed to explain the maximum amount of variability in the data. Model 1 demonstrated significantly increased odds of seropositivity in mares. On descriptive analyses, statistically significant associations were measured between sex and variables pertaining to, work status, use for transport or construction purposes, travel to Senegal, being allowed to roam versus being tethered, and grazing activity; thus, sex should be explored as a potential confounder. The effect of these management variations on the odds of *Histoplasma* spp. exposure and temporal trends in engagement in work, including for farming purposes, warrant further investigation. A greater proportion of stallions were engaged in work during the dry season and used for transport or construction activities. Whereas a greater proportion of mares were loose during the day and allowed to graze for food during the dry season. Increased aerosolisation and inhalation of fungal elements, associated with grazing or free-roaming behaviours (and facilitated by climatic conditions during the dry season), are theoretically plausible explanations for increased *Histoplasma* spp. exposure in mares. Systematic sampling of environments frequented by mares is warranted. Furthermore, the impacts of gestation and changes in BCS on immunocompetence amongst mares warrant investigation. Although evidence of associations between mare reproductive status and *Histoplasma* spp. infection status is lacking, transplacental *Histoplasma* transmission and postpartum histoplasmosis have been reported in the human literature (44, 45).

Both models demonstrated decreased odds of seropositivity amongst horses <2.5 years, compared to horses ≥4.5 years. A lower proportion of horses <2.5 years were used for ploughing or engaged in work during the rainy season, compared to animals aged ≥4.5 years. Involvement in farming activities by older age groups may increase their odds of *Histoplasma* spp. exposure, in association with increased disturbance of *Histoplasma* spp. soil reservoirs by ploughing, increased occurrence of harness-related wounds facilitating fungal inoculation of cutaneous abrasions, or immunosuppression related to engagement in farming activities. Although harnesses are designed specifically for equid species in the Gambian context (rather than individual harnesses being used on all draught animals, including oxen), the same harness can be used by multiple horses or donkeys (DLS, personal communication). Thus, sharing of harnesses, and wounds related to poor harness fit, may facilitate *Histoplasma* spp. transmission and exposure. Harness-related wounds and inoculation of cutaneous abrasions with *Histoplasma* spp. elements have been explored as routes of infection (14, 46). The observation of non-EL wounds on clinical examination was not significantly associated with *Histoplasma* serostatus or infection status on univariable analysis, either for horse or donkey study populations. The study populations were asymptomatic for EL; thus, routes of exposure and infection may differ from those described for the clinical forms of the disease. Furthermore, a focussed investigation of age-related immunocompetence in relation to anti-*Histoplasma* antibody response is warranted. Although a higher prevalence of EL has been measured in older working equids >10 years in Ethiopia, no significant association between age category and odds of infection was measured on univariable logistic regression analysis (28). To date, cross-sectional studies exploring risk factors for EL have primarily been conducted in Ethiopia and used univariable analysis (28, 40, 41). Thus, additional multivariable regression analyses are warranted across diverse contexts to examine the complex interplay of demographic, clinical, and environmental factors associated with *Histoplasma* spp. exposure and infection, and to identify potential confounding variables (38). The age distribution of horses ≥4.5 years is unknown, which limits accurate examination of age-related immunocompetence in these animals. Investigation of age-associated immunosuppression on IgM response to *Histoplasma* spp., related to physiological stress or inflammatory responses, is warranted.

Model 2 demonstrated significantly increased odds of seropositivity in horses during the rainy season, which concurs with anecdotal evidence describing seasonal trends in EL cases in The Gambia (DLS, personal communication). Geoclimatic factors have been explored as risk factors for clinical EL in working horses in Ethiopia by univariable (47) and multivariable analysis (39). The Gambia demonstrates seasonal variations in rainfall, humidity, and temperature. Both multivariable models relating to *Histoplasma* spp. seropositivity in horses demonstrated low variance by study site. Except for two regions, the majority of study animals were recruited during the dry season, and an association was measured between season and region (χ^2^-associated *p* < 0.01). The Gambia demonstrates four agroecological zones with distinctive geoclimatic characteristics (48), seasonal variations in rainfall, humidity, and temperature, and an average elevation above sea level of 34 metres (range 0–53 metres). The impact of seasonal and regional climatic variations on the survival of *Histoplasma* spp. mycelial form and the odds of equid *Histoplasma* spp. exposure warrant further investigation, as described in other contexts (39, 47). The association between environmental conditions and HCF viability in soil and water has been demonstrated experimentally (49), whilst physical and chemical profiles of soil and avian faeces that might favour the survival of *Histoplasma* spp. mycelia have been examined (50–52). Thus, the impact of variations in the physical and chemical characteristics of soil in The Gambia in relation to region, season, or proximity to water courses (including the River Gambia) should be examined. Longitudinal research is warranted to measure temporal trends in *Histoplasma* spp. exposure and equid management strategies.

Model 2 presented significantly increased odds of seropositivity in horses belonging to households reporting previous cutaneous EL cases in any owned equid. Investigation of ecological niches for *Histoplasma* spp. in the compound setting is warranted, including systematic environmental sampling. Potential *Histoplasma* spp. reservoirs within the Gambian compound may comprise deep piles of animal manure mixed with soil (which can be spread on the ground where equids are managed) or poultry housing. These hypotheses are supported by human literature describing statistically significant increased odds of *Histoplasma* spp. seropositivity amongst humans involved in animal manure management in The Gambia (53). Previous EL cases were owner-reported, limited to the cutaneous presentation, and unconfirmed by definitive diagnostics; however, this variable may be a useful predictor of *Histoplasma* spp. maintenance in the compound.

Model 2 demonstrated significantly lower odds of seropositivity amongst horses used to transport firewood, contrary to the expected direction of association based on evidence of clinical EL occurrence in cart horses in other settings (39). When horses are engaged for transportation purposes within the Gambian context, one could hypothesise that they are less likely to be engaged in farming activities; thus, this outcome might reflect reduced exposure to farming-related risk factors for *Histoplasma* spp. exposure. Although equid contributions to farming during the rainy season have been described (DLS, personal communication), investigation of seasonal variations in equid uses for *transportation* purposes is warranted. On multivariable analyses, sex, age, season, and region were explored as fixed effects and demonstrated associations with variables describing equid uses. Further investigation may disentangle individual and cumulative effects of these variables on *Histoplasma* spp. exposure.

The apparent increased clinical severity of EL in horses and their higher socioeconomic status compared to donkeys may have contributed to horse populations being the focus of previous EL research. The donkey population here demonstrated significantly lower odds of both *Histoplasma* spp. seropositivity and HCF infection, which concurs with current literature describing higher infection prevalence in horses (28, 54).

Notably, the donkey study population demonstrated decreased odds of seropositivity in animals from households that also owned horses. This may reflect the differential uses of horses and donkeys in multi-equid compounds. Based on anecdotal reports, farmers will preferentially use horses for farm work during the rainy season as opposed to donkeys (if both are available); thus, one can hypothesise that if horses are owned, the donkey is less likely to be used for farming, leading to decreased exposure to *Histoplasma* spp. in relation to soil disturbance during ploughing, harness-related wound occurrence or physiological stress (DLS, personal communication). Furthermore, if both horses and donkeys are available, donkeys are more likely to be used for the transportation of people, goods, and firewood. One might expect more frequent *Histoplasma* spp. exposure amongst donkeys from households with horses due to the increased EL occurrence and severity reported in horses by our study and in the literature (28, 55). Although this does not negate the argument of differential use of horses and donkeys impacting odds of seropositivity, focussed examination of the main clinical and management-related predictors for *Histoplasma* spp. exposure, in donkeys managed with and without horses, is warranted. Equid species-specific variations in pathophysiology and immunological and inflammatory responses to *Histoplasma* spp. also warrant investigation.

The evidence presented by this study indicates that *Histoplasma* spp. are endemic across The Gambia suggesting that environmental conditions are conducive to the proliferation and survival of the saprophytic mycelial form. Economic and sociocultural factors may prohibit the culling of infected animals (13, 15). Historical environmental decontamination strategies have been described, including the burning of equine bedding and burial of surface soil with quick lime in the UK (56) and the use of formalin to disinfect bird roosts and soil in response to human histoplasmosis outbreaks (57, 58). Although intensive decontamination strategies may not be ruled out in cases of severe outbreaks of EL in a defined and restricted geographic area, these practices may be contra-indicated in a community setting in The Gambia due to the adverse health and environmental effects of decontamination strategies and limited access to personal protective equipment and healthcare services. The risk factors highlighted by the multivariable risk factor analyses should be explored to understand how variable equid management strategies by age, sex, and season impact the risk of *Histoplasma* spp. exposure or infection. These findings, in addition to educational programmes on infectious disease control and biosecurity practices, should inform context-specific and practical EL prevention and control strategies. Furthermore, the impact, feasibility, and sustainability of recommendations to reduce the burden of *Histoplasma* spp., including equid or environmental management variations or household behavioural changes, should be assessed.

### 4.2 Study limitations

Only study sites with relatively high equid populations were selected. Previous evidence describes increased EL occurrence in areas of high equid populations and congregation sites (47); thus, our estimations of *Histoplasma* spp. exposure prevalence may not be representative of areas with lower equid populations.

The LAT provided a non-quantitative assessment of anti-*Histoplasma* antibody presence and only one LAT was performed per serum sample. To provide an estimation of antibody titre, serum dilutions could be performed. Detection of HCF on nested ITS-PCR provided additional evidence to support a seropositive result, although depending on relative timings of seroconversion and development of fungemia, a seropositive but PCR-negative outcome does not negate positive exposure. Furthermore, the LAT can cross-react with other endemic mycoses, which could lead to false-positive reactions (59). Evidence of pathogenic fungi in The Gambia comprises historic human case reports describing *Histoplasma* spp. (60–62) and *Blastomyces* spp. (63), and an estimation of superficial fungal infection prevalence (64). Furthermore, *Aspergillus*-associated aflatoxins have been implicated as a major crop contaminant in the Gambian context with significant health implications (65). Equid exposure to these fungal species may result in LAT cross-reactions.

Due to the preferential presentation of horses and significantly lower prevalence of *Histoplasma* spp. exposure and infection in donkeys, variables under examination may not have been sufficiently powered to explore as risk factors. Thus, focussed exploration of *Histoplasma* spp. burden and associated risk factors in a larger sample of donkeys is warranted.

Clinical samples were temporarily stored and transported between field sites and laboratories. Inconsistent electricity supply at DLS Regional Offices and movement during transport might have decreased sample integrity. The significant challenge of maintaining the cold chain from sample collection to processing and the potential impact on LAT results and HCF detection should not be underestimated. These effects were reduced by identifying sites with backup generators, strict monitoring of the electricity supply, and careful transport.

## 5 Conclusion

The study provides evidence of high anti-*Histoplasma* antibody seroprevalence in horse and donkey populations in The Gambia and a wide geographic distribution of seropositive animals, which supports the endemic status of the country with respect to *Histoplasma* spp. Furthermore, evidence of asymptomatic carriage of *Histoplasma* spp. in apparently healthy animals is provided.

The cutaneous form of EL in low-resource settings has been the focus of previous literature due to its significant socioeconomic impact associated with reduced equid working capacity (66). Longitudinal analysis is warranted to explore temporal trends in *Histoplasma* spp. exposure *and* infection, and the effect of geoclimatic, demographic, clinical, and management variations on the risk of clinical disease development and progression, including the manifestation of cutaneous, respiratory, and ocular forms of the disease.

The following areas warrant further investigation in working equids in rural The Gambia: (i) the individual and potentially cumulative effect of highlighted clinical, management, and environmental risk factors on odds of equid *Histoplasma* spp. exposure, infection, and development of clinical disease; (ii) *Histoplasma* spp. transmission dynamics in the compound, including the respective roles of horses and donkeys in *Histoplasma* spp. environmental maintenance; (iii) systematic sampling of potential *Histoplasma* spp. reservoirs, in relation to farm and compound settings; and (iv) comparative phylogenetic analysis of *Histoplasma* spp. isolates of human, equid, and environmental origins. In line with a One Health approach, these areas of investigation are warranted to examine *Histoplasma* spp. transmission dynamics at this human-animal-environment interface and to explore the public health implications of environmental and animal reservoirs of *Histoplasma* spp. in this setting.

Study outcomes and proposed research areas will inform the development of sustainable EL control strategies in The Gambia and research directions across comparable settings worldwide to ascertain the importance of described risk factors in the global epidemiology of *Histoplasma* spp.

## Data Availability

The raw data supporting the conclusions of this article will be made available by the authors, without undue reservation.
