# Supplementary material for: Exploring Histoplasma species seroprevalence and risk factors for seropositivity in The Gambia’s working equid population: Baseline analysis of the Tackling Histoplasmosis project dataset
Source: Front Vet Sci. 2024 Sep 19;11:1444887. doi: 10.3389/fvets.2024.1444887 (PMC11446873; doi:10.3389/fvets.2024.1444887)
Supplement: Supplementary file 1 [file Table_S1.docx]

**S1 Table.** Frequency distributions of horse (*N*=463) and donkey (*N*=92) study populations, and *Histoplasma* spp. seropositivity based on Latex Agglutination Test (LAT) result, by region, study site and season in The Gambia.

|  | **HORSES, *N*=463** | | **DONKEYS, *N*=92** | | **TOTAL EQUIDS, *N*=555** | |
| --- | --- | --- | --- | --- | --- | --- |
| **Variable** | **Frequency, *n* (%)** | **Seropositive, *n* (%)** | **Frequency, *n* (%)** | **Seropositive, *n* (%)** | **Frequency, *n* (%)** | **Overall seropositive, *n* (%)** |
| **Recruitment region and study site** | | | | | | |
| **Upper River Region** | 134 (28.9) | 113 (84.3) ^a^ | 22 (23.9) | 13 (59.1) | 156 (28.1) | 126 (80.8) ^a^ |
| BAJ | 30 (6.5) | 22 (73.3) ^a^ | 0 (0.0) | - | 30 (5.4) | 22 (73.3) ^a^ |
| DIN | 29 (6.3) | 25 (86.2) | 0 (0.0) | - | 29 (5.2) | 25 (86.2) |
| GAR | 29 (6.3) | 27 (93.1) | 0 (0.0) | - | 29 (5.2) | 27 (93.1) |
| SAB | 28 (6.0) | 23 (82.1) | 10 (10.9) | 4 (40.0) | 38 (6.8) | 27 (71.1) |
| SNG | 18 (3.9) | 16 (88.9) | 12 (13.0) | 9 (75.0) | 30 (5.4) | 25 (83.3) |
| **Central River Region/ North** | 99 (21.4) | 72 (72.7) | 1 (1.1) | 0 (0.0) | 100 (18.0) | 72 (72.0) |
| NDO | 41 (8.9) | 33 (80.5) | 0 (0.0) | - | 41 (7.4) | 33 (80.5) |
| NGU ^b^ | 28 (6.0) | 23 (82.1) | 1 (1.1) | 0 (0.0) | 29 (5.2) | 23 (79.3) |
| WAS | 30 (6.5) | 16 (53.3) | 0 (0.0) | - | 30 (5.4) | 16 (53.3) |
| **Central River Region/ South** | 68 (14.7) | 51 (75.0) | 19 (20.7) | 3 (15.8) | 87 (15.7) | 54 (62.1) |
| BRI ^b^ | 26 (5.6) | 22 (84.6) | 1 (1.1) | 0 (0.0) | 27 (4.9) | 22 (81.5) |
| GAL | 30 (6.5) | 24 (80.0) | 0 (0.0) | - | 30 (5.4) | 24 (80.0) |
| JAR | 12 (2.6) | 5 (41.7) | 18 (19.6) | 3 (16.7) | 30 (5.4) | 8 (26.7) |
| **North Bank Region** | 89 (19.2) | 75 (84.3) | 32 (34.8) | 15 (46.9) | 121 (21.8) | 90 (74.4) |
| CJI | 19 (4.1) | 18 (94.7) | 11 (12.0) | 5 (45.5) | 30 (5.4) | 23 (76.7) |
| KPK ^b^ | 19 (4.1) | 15 (78.9) | 8 (8.7) | 4 (50.0) | 27 (4.9) | 19 (70.4) |
| MSM | 30 (6.5) | 26 (86.7) | 0 (0.0) | - | 30 (5.4) | 26 (86.7) |
| NSA ^b^ | 21 (4.5) | 16 (76.2) | 13 (14.1) | 6 (46.2) | 34 (6.1) | 22 (64.7) |
| **Lower River Region** | 60 (13.0) | 48 (80.0) | 1 (1.1) | 1 (100.0) | 61 (11.0) | 49 (80.3) |
| MAD | 32 (6.9) | 26 (81.3) | 0 (0.0) | - | 32 (5.8) | 26 (81.3) |
| SBJ | 28 (6.0) | 22 (78.6) | 1 (1.1) | 1 (100.0) | 29 (5.2) | 23 (79.3) |
| **West Coast Region** | 13 (2.8) | 11 (84.6) | 17 (18.5) | 11 (64.7) | 30 (5.4) | 22 (73.3) |
| BAM ^b^ | 13 (2.8) | 11 (84.6) | 17 (18.5) | 11 (64.7) | 30 (5.4) | 22 (73.3) |
| **Total animals, *n*** | 463 (100.00) | 370 (79.9) | 92 (100.00) | 43 (46.7) | 555 (100.00) | 413 (74.4) |
| **Season of recruitment** | | | | | | |
| Dry (November- May) | 325 (70.2) | 252 (77.5) ^a^ | 40 (43.5) | 19 (47.5) | 365 (65.8) | 271 (74.2) |
| Rainy (June- October) | 138 (29.8) | 118 (85.5) | 52 (56.5) | 24 (46.2) | 190 (34.2) | 142 (74.7) |

^a^ Missing serum sample for *n*=1 horse (no LAT result); ^b^ Two study recruitment locations per study site.
