# Supplementary material for: Exploring Histoplasma species seroprevalence and risk factors for seropositivity in The Gambia’s working equid population: Baseline analysis of the Tackling Histoplasmosis project dataset
Source: Front Vet Sci. 2024 Sep 19;11:1444887. doi: 10.3389/fvets.2024.1444887 (PMC11446873; doi:10.3389/fvets.2024.1444887)
Supplement: Supplementary file 2 [file Table_S2.docx]

**S2 Table.** Demographic and baseline clinical characteristics of horse (*N*=463) and donkey (*N*=92) study populations in The Gambia.

|  | | HORSES, *N*=463 | DONKEYS, *N*=92 |
| --- | --- | --- | --- |
| Variable | | **Frequency, n (%)** | |
| Demographic |  | | |
| Sex | |  |  |
| Male | | 259 (55.9) | 43 (46.7) |
| Female | | 204 (44.1) | 49 (53.3) |
| Age, years ^a^ | |  |  |
| <2.5^H^/ <3.5^D^ | | 44 (9.5) | 5 (5.4) |
| 2.5-4.5^H^/ 3.5-5.5^D^ | | 46 (9.9) | 8 (8.7) |
| ≥4.5^H^/ ≥5.5^D^ | | 373 (80.6) | 79 (85.9) |
| Reproductive history ^b^ | | | |
| Currently in foal | |  |  |
| No | | 83 (17.9) | 26 (28.3) |
| Yes | | 28 (6.0) | 10 (10.9) |
| NR/ND | | 93 (20.1) | 13 (14.1) |
| NA (male) | | 259 (55.9) | 43 (46.7) |
| Previously in foal | |  |  |
| No | | 59 (12.7) | 20 (21.7) |
| Yes | | 50 (10.8) | 16 (17.4) |
| NR/ND or currently in foal | | 95 (20.5) | 13 (14.1) |
| NA (male) | | 259 (55.9) | 43 (46.7) |
| Clinical examination |  | | |
| General demeanour | |  |  |
| BAR | | 440 (95.0) | 88 (95.7) |
| QAR | | 18 (3.9) | 2 (2.2) |
| Dull | | - | 1 (1.1) |
| NR/ND | | 5 (1.1) | 1 (1.1) |
| BCS, 0-5 | |  |  |
| Median (IQR) | | 3.0 (2.0-3.0) | 3.0 (3.0-3.0) |
| MM colour | |  |  |
| Pale pink | | 443 (95.7) | 87 (94.6) |
| White | | 17 (3.7) | 5 (5.4) |
| NR/ND | | 3 (0.6) | - |
| MM hydration | |  |  |
| Moist | | 393 (84.9) | 76 (82.6) |
| Tacky | | 63 (13.6) | 15 (16.3) |
| NR/ND | | 7 (1.5) | 1 (1.1) |
| CRT, seconds | |  |  |
| <2 | | 422 (91.1) | 85 (92.4) |
| 2 | | 35 (7.6) | 7 (7.6) |
| >2 | | 2 (0.4) | 0 (0.0) |
| NR/ND | | 4 (0.9) | - |
| Ocular examination |  | | |
| Ocular discharge | |  |  |
| No | | 319 (68.9) | 81 (88.0) |
| Yes | | 140 (30.2) | 11 (12.0) |
| NR/ND | | 4 (0.9) | - |
| Ocular discharge character | |  |  |
| No ocular discharge | | 319 (68.9) | 81 (88.0) |
| Serous (+/- mucoid) | | 58 (12.5) | 7 (7.6) |
| Mucoid | | 60 (13.0) | 4 (4.3) |
| Mucopurulent (+/- serous or mucoid) | | 22 (4.8) | 0 (0.0) |
| Sanguineous | | 0 (0.0) | 0 (0.0) |
| NR/ND | | 4 (0.9) | - |
| Ocular pathology | |  |  |
| No | | 420 (90.7) | 91 (98.9) |
| Yes | | 40 (8.6) | 1 (1.1) |
| NR/ND | | 3 (0.6) | - |
| Peri-orbital pathology | |  |  |
| No | | 415 (89.6) | 89 (96.7) |
| Yes | | 45 (9.7) | 3 (3.3) |
| NR/ND | | 3 (0.6) | - |
| Lymph node palpation |  | | |
| Submandibular | |  |  |
| Within normal limits | | 390 (84.2) | 88 (95.7) |
| Enlarged | | 68 (14.7) | 4 (4.3) |
| Abscessated | | 2 (0.4) | 0 (0.0) |
| Unable to palpate | | 0 (0.0) | 0 (0.0) |
| NR/ND | | 3 (0.6) | - |
| Retropharyngeal | |  |  |
| Within normal limits | | 449 (97.0) | 92 (100.0) |
| Enlarged | | 11 (2.4) | 0 (0.0) |
| Abscessated | | 0 (0.0) | 0 (0.0) |
| Unable to palpate | | 0 (0.0) | 0 (0.0) |
| NR/ND | | 3 (0.6) | - |
| Prescapular | |  |  |
| Within normal limits | | 456 (98.5) | 92 (0.0) |
| Enlarged | | 4 (0.9) | 0 (0.0) |
| Abscessated | | 0 (0.0) | 0 (0.0) |
| Unable to palpate | | 0 (0.0) | 0 (0.0) |
| NR/ND | | 3 (0.6) | - |
| Popliteal | |  |  |
| Within normal limits | | 399 (86.2) | 78 (84.8) |
| Enlarged | | 1 (0.2) | 0 (0.0) |
| Abscessated | | 0 (0.0) | 0 (0.0) |
| Unable to palpate | | 5 (1.1) | 1 (1.1) |
| NR/ND | | 58 (12.5) | 13 (14.1) |
| Respiratory examination |  | | |
| Nasal discharge | |  |  |
| No | | 283 (61.1) | 76 (82.6) |
| Yes | | 176 (38.0) | 16 (17.4) |
| NR/ND | | 4 (0.9) | - |
| Nasal discharge character | |  |  |
| No nasal discharge | | 283 (61.1) | 76 (82.6) |
| Serous (+/- mucoid) | | 171 (36.9) | 16 (17.4) |
| Mucopurulent (+/- serous) | | 5 (1.1) | 0 (0.0) |
| Sanguineous | | 0 (0.0) | 0 (0.0) |
| Epistaxis | | 0 (0.0) | 0 (0.0) |
| NR/ND | | 4 (0.9) | - |
| Increased or abnormal respiratory sounds ^c^ | |  |  |
| No | | 457 (98.7) | 90 (97.8) |
| Increased normal | | 1 (0.2) | 1 (1.1) |
| NR/ND | | 5 (1.1) | 1 (1.1) |
| Increased or abnormal tracheal sounds | |  |  |
| No | | 344 (74.3) | 63 (68.5) |
| Increased normal | | 114 (24.6) | 29 (31.5) |
| Decreased normal | | 1 (0.2) | - |
| Wheeze | | 1 (0.2) | - |
| NR/ND | | 4 (0.9) | - |
| Increased or abnormal thoracic sounds | |  |  |
| No | | 375 (81.0) | 72 (78.3) |
| Increased normal | | 62 (13.4) | 9 (9.8) |
| Wheeze | | 4 (0.9) | 1 (1.1) |
| Crackles | | 1 (0.2) | - |
| NR/ND | | 21 (4.5) | 10 (10.9) |
| Gastrointestinal auscultation | | | |
| Left dorsal quadrant | |  |  |
| Normal | | 325 (70.2) | 55 (59.8) |
| Decreased | | 70 (15.1) | 30 (32.6) |
| Increased | | 63 (13.6) | 7 (7.6) |
| Absent | | 2 (0.4) | 0 (0.0) |
| NR/ND | | 3 (0.6) | - |
| Left ventral quadrant | |  |  |
| Normal | | 322 (69.5) | 63 (68.5) |
| Decreased | | 61 (13.2) | 20 (21.7) |
| Increased | | 75 (16.2) | 9 (9.8) |
| Absent | | 2 (0.4) | 0 (0.0) |
| NR/ND | | 3 (0.6) | - |
| Right dorsal quadrant | |  |  |
| Normal | | 338 (73.0) | 55 (59.8) |
| Decreased | | 66 (14.3) | 28 (30.4) |
| Increased | | 54 (11.7) | 8 (8.7) |
| Absent | | 1 (0.2) | 1 (1.1) |
| NR/ND | | 4 (0.9) | - |
| Right ventral quadrant | |  |  |
| Normal | | 332 (71.7) | 58 (63.0) |
| Decreased | | 53 (11.4) | 18 (19.6) |
| Increased | | 74 (16.0) | 15 (16.3) |
| Absent | | 0 (0.0) | 1 (1.1) |
| NR/ND | | 4 (0.9) | - |
| Musculoskeletal examination | | | |
| Lameness ^b^ | |  |  |
| No | | 413 (89.2) | 83 (90.2) |
| Yes | | 38 (8.2) | 8 (8.7) |
| NR/ND | | 12 (2.5) | 1 (1.1) |
| Limb swelling | |  |  |
| No | | 423 (91.4) | 91 (98.9) |
| Yes | | 34 (7.3) | 1 (1.1) |
| NR/ND | | 6 (1.3) | - |
| Digital pulse palpation | |  |  |
| Left forelimb | |  |  |
| Not palpable | | 281 (60.7) | 72 (78.3) |
| Palpable | | 166 (35.9) | 20 (21.7) |
| Bounding | | 11 (2.4) | 0 (0.0) |
| NR/ND | | 5 (1.1) | - |
| Left hindlimb | |  |  |
| Not palpable | | 300 (64.8) | 81 (88.0) |
| Palpable | | 114 (24.6) | 5 (5.4) |
| Bounding | | 20 (4.3) | 0 (0.0) |
| NR/ND | | 29 (6.3) | 6 (6.5) |
| Right forelimb | |  |  |
| Not palpable | | 277 (59.8) | 73 (79.3) |
| Palpable | | 169 (36.5) | 18 (19.6) |
| Bounding | | 12 (2.6) | 0 (0.0) |
| NR/ND | | 5 (1.1) | 1 (1.1) |
| Right hindlimb | |  |  |
| Not palpable | | 283 (61.1) | 80 (87.0) |
| Palpable | | 112 (24.2) | 4 (4.3) |
| Bounding | | 24 (5.2) | 0 (0.0) |
| NR/ND | | 44 (9.5) | 8 (8.7) |
| Total digital pulses palpable, number | |  |  |
| Median (IQR) | | 1.0 (0.0-2.0) | 0.0 (0.0-1.0) |
| Abnormal hoof conformation | |  |  |
| No | | 143 (30.9) | 32 (34.8) |
| Yes | | 317 (68.5) | 60 (65.2) |
| NR/ND | | 3 (0.6) | - |
| Abnormal hoof conformation: Type | |  |  |
| *Long toe(s)* | |  |  |
| No | | 240 (51.8) | 50 (54.3) |
| Yes | | 220 (47.5) | 42 (45.7) |
| NR/ND | | 3 (0.6) | - |
| *Boxy hoof/hooves* | |  |  |
| No | | 426 (92.0) | 86 (93.5) |
| Yes | | 34 (7.3) | 6 (6.5) |
| NR/ND | | 3 (0.6) | - |
| *Low heel(s)* | |  |  |
| No | | 236 (51.0) | 67 (72.8) |
| Yes | | 224 (48.4) | 25 (27.2) |
| NR/ND | | 3 (0.6) | - |
| *Hoof wall crack(s) or chip(s)* | |  |  |
| No | | 387 (83.6) | 70 (76.1) |
| Yes | | 73 (15.8) | 22 (23.9) |
| NR/ND | | 3 (0.6) | - |
| *Flared hoof wall(s)* | |  |  |
| No | | 415 (89.6) | 92 (100.0) |
| Yes | | 45 (9.7) | 0 (0.0) |
| NR/ND | | 3 (0.6) | - |
| Hoof management ^b^ | | | |
| Hoof maintenance ^d^ | |  |  |
| No | | 33 (7.1) | 4 (4.3) |
| Yes | | 430 (92.9) | 86 (93.5) |
| NR/ND | | - | 2 (2.2) |
| Owner trims hooves | |  |  |
| No | | 409 (88.3) | 64 (69.6) |
| Yes | | 52 (11.2) | 23 (25.0) |
| NR/ND | | 2 (0.4) | 5 (5.4) |
| Farrier ^e^ trims hooves | |  |  |
| No | | 323 (69.8) | 79 (85.9) |
| Yes | | 138 (29.8) | 8 (8.7) |
| NR/ND | | 2 (0.4) | 5 (5.4) |
| Owner monitors hoof shape | |  |  |
| No | | 40 (8.6) | 4 (4.3) |
| Yes | | 421 (90.0) | 83 (90.2) |
| NR/ND | | 2 (0.4) | 5 (5.4) |
| Ectoparasite management |  | | |
| Types | |  |  |
| No ectoparasites | | 84 (18.1) | 72 (78.3) |
| Ticks | | 343 (74.1) | 19 (20.7) |
| Flies | | 5 (1.1) | 1 (1.1) |
| Flies and ticks | | 26 (5.6) | - |
| NR/ND | | 5 (1.1) | - |
| Treatment ^b^ | |  |  |
| No | | 240 (51.8) | 60 (65.2) |
| Yes | | 207 (44.7) | 30 (32.6) |
| NR/ND | | 16 (3.5) | 2 (2.2) |
| Endoparasite management ^b^ | | | |
| Treatment | |  |  |
| No | | 70 (15.1) | 28 (30.4) |
| Yes | | 393 (84.9) | 62 (67.4) |
| NR/ND | | - | 2 (2.2) |
| Anthelmintic drugs from vet pharmacy | |  |  |
| No | | 186 (40.2) | 40 (43.5) |
| Yes | | 277 (59.8) | 50 (54.3) |
| NR/ND | | - | 2 (2.2) |
| Anthelmintic drugs from other sources | |  |  |
| No | | 246 (53.1) | 59 (64.1) |
| Yes | | 217 (46.9) | 31 (33.7) |
| NR/ND | | - | 2 (2.2) |
| Anthelmintic drugs from other sources: Specify | |  |  |
| LA | | 177 (38.2) | 23 (25.0) |
| *Lumo*/ local trader | | 21 (4.5) | 4 (4.3) |
| LA and local trader | | 6 (1.3) | - |
| LA and GHDT | | 1 (0.2) | - |
| Don’t administer from other sources | | 246 (53.1) | 59 (64.1) |
| NR/ND | | 12 (2.6) | 6 (6.5) |
| Wounds (not Epizootic Lymphangitis) | | | |
| Wounds | |  |  |
| No | | 357 (77.1) | 60 (65.2) |
| Yes | | 103 (22.2) | 32 (34.8) |
| NR/ND | | 3 (0.6) | - |
| Wound treatment ^b^ | |  |  |
| No wounds | | 357 (77.1) | 60 (65.2) |
| No | | 59 (12.7) | 26 (28.3) |
| Yes | | 39 (8.4) | 6 (6.5) |
| NR/ND | | 8 (1.7) | - |
| Epizootic Lymphangitis history ^b^ | | | |
| Previous EL | |  |  |
| No | | 324 (70.0) | 76 (82.6) |
| Yes | | 138 (29.8) | 14 (15.2) |
| NR/ND | | 1 (0.2) | 2 (2.2) |
| Current EL in other owned equid(s) ^f^ | |  |  |
| No | | 458 (98.9) | 89 (96.7) |
| Yes | | 4 (0.9) | 1 (1.1) |
| NR/ND | | 1 (0.2) | 2 (2.2) |

BAR=Bright alert responsive; QAR=Quiet alert responsive; BCS=Body Condition Score; MM=Mucous Membranes; CRT=Capillary Refill Time (on palpation of oral mucosa); EL=Epizootic Lymphangitis; LA=Livestock Agent; GHDT=Gambia Horse and Donkey Trust; NA=Not applicable; NR/ND=No response/ No data.

^H^ Horse; ^D^ Donkey. ^a^ Age estimated based on incisor eruption times; ^b^ Owner-reported variables; ^c^ “Increased or abnormal respiratory sounds” refer to increased or abnormal respiratory sounds when standing next to animal; ^d^ “Hoof maintenance” includes the following: owner monitors foot and hoof shape, owner trims hooves, owner visits farrier to trim hooves, or other specified hoof management described by owner; ^e^ “Farriers” are defined as individuals with basic training in farriery (these are not the Livestock Agents and they may have other sources of incomes, such as farming); ^f^ Current Epizootic Lymphangitis reported by owner in other owned equids excluding the study animal.
