# Supplementary material for: Exploring Histoplasma species seroprevalence and risk factors for seropositivity in The Gambia’s working equid population: Baseline analysis of the Tackling Histoplasmosis project dataset
Source: Front Vet Sci. 2024 Sep 19;11:1444887. doi: 10.3389/fvets.2024.1444887 (PMC11446873; doi:10.3389/fvets.2024.1444887)
Supplement: Supplementary file 3 [file Table_S3.docx]

**S3 Table.** Baseline characteristics of horse (*N*=463) and donkey (*N*=92) study populations in The Gambia, pertaining to haematological and biochemical parameters.

|  | **HORSES, *N*=463** | | **DONKEYS, *N*=92** | |
| --- | --- | --- | --- | --- |
|  | Dry (*n*=325) | Rainy (*n*=138) | Dry (*n*=40) | Rainy (*n*=52) |
| **Variable** | **Median (IQR)** | | | |
| **Packed Cell Volume, %** | 30.0 (25.8-34.0) | 30.0 (25.0-34.0) | 33.0 (30.0-37.0) | 31.0 (28.0-34.0) |
| **Total Protein, g/L** | 80.0 (72.0-86.0) | 73.5 (70.0-80.0)* | 78.0 (70.3-82.0) | 80.0 (72.0-82.0) |
| **Neutrophil, %** | 42.0 (20.0-54.9) | 25.0 (17.5-40.1)* | 26.2 (16.8-39.2) | 22.1 (16.5-30.0) |
| **Eosinophil, %** | 5.0 (3.5-7.5) | 7.0 (5.0-9.8)* | 5.4 (4.1-7.0) | 6.8 (5.0-9.0)* |
| **Basophil, %** | 1.0 (0.5-2.0) | 1.0 (0.0-1.8) | 1.5 (0.5-2.5) | 1.0 (0.49-2.0) |
| **Monocyte, %** | 0.5 (0.0-1.3) | 0.0 (0.0-0.5)* | 1.0 (0.0-2.5) | 0.0 (0.0-0.9)* |
| **Lymphocyte, %** | 50.0 (37.5-68.9) | 64.5 (48.8-74.5)* | 60.0 (51.9-70.0) | 69.3 (60.0-75.4) |

* *U*-test *p*<0.05.
