# Supplementary material for: Exploring Histoplasma species seroprevalence and risk factors for seropositivity in The Gambia’s working equid population: Baseline analysis of the Tackling Histoplasmosis project dataset
Source: Front Vet Sci. 2024 Sep 19;11:1444887. doi: 10.3389/fvets.2024.1444887 (PMC11446873; doi:10.3389/fvets.2024.1444887)
Supplement: Supplementary file 4 [file Table_S4.docx]

**S4 Table.** Locations of wounds (not associated with Epizootic Lymphangitis) in study horses (*n*=103/463, 22.2%) and donkeys (*n*=31/92, 33.7%), and owner-reported wound treatments.

|  | | HORSES, *n*=103/463 | DONKEYS, *n*=31/92 |
| --- | --- | --- | --- |
| Variable | | **Frequency, n (%)** | |
| Wound location |  | | |
| Tail or dock | | 5 (4.5) | 9 (23.7) |
| Chest | | 8 (7.1) | - |
| Back or withers | | 19 (17.0) | 3 (7.9) |
| Shoulder or neck | | 16 (14.3) | 10 (26.3) |
| Elbow | | 4 (3.6) | - |
| Forelimb(s) ^b^ | | 11 (9.8) | 3 (7.9) |
| Hindlimb(s) | | 22 (19.6) | 4 (10.5) |
| Head | | 12 (10.7) | 7 (18.4) |
| Point of hip | | 5 (4.5) | 1 (2.6) |
| Flank or barrel | | 5 (4.5) | 1 (2.6) |
| Unspecified | | 5 (4.5) | - |
| Total wounds, *n* | | 112 | 38 |
| Owner-reported wound treatment | | | |
| No treatment | | 59 (57.3) | 25 (80.6) |
| Treatment unknown or unspecified treatment by Livestock Agent | | 12 (11.7) | - |
| Topical antibiotic | | 9 (8.7) | - |
| Antibiotic, *per os* or intramuscular | | 2 (1.9) | - |
| Anti-inflammatory | | 1 (1.0) | - |
| “Burn oil” or engine oil | | 15 (14.6) | 3 (9.7) |
| Battery acid | | 2 (1.9) | - |
| Dettol | | 1 (1.0) | 1 (3.2) |
| Plant | | 1 (1.0) ^c^ | 2 (6.5) ^d^ |
| Unspecified | | 1 (1.0) | - |

^a^ Chest wounds (*n*=8) were superficial without evidence of infection; ^b^ Distal to point of elbow; ^c^ “Leaf of *bembe*” ground up and applied topically; ^d^ “Local treatment called *fudan*” (natural dye from leaf).
