# Supplementary material for: Exploring Histoplasma species seroprevalence and risk factors for seropositivity in The Gambia’s working equid population: Baseline analysis of the Tackling Histoplasmosis project dataset
Source: Front Vet Sci. 2024 Sep 19;11:1444887. doi: 10.3389/fvets.2024.1444887 (PMC11446873; doi:10.3389/fvets.2024.1444887)
Supplement: Supplementary file 5 [file Table_S5.docx]

**S5 Table.** Baseline characteristics of horse (*N*=463) and donkey (*N*=92) study populations in The Gambia, pertaining to animal management and environmental factors.

|  | **HORSES, *N*=463** | **DONKEYS, *N*=92** |
| --- | --- | --- |
| **Variable** | **Frequency, *n* (%)** | |
| **Equid role** | | |
| **Ploughing** |  |  |
| No | 29 (6.3) | 6 (6.5) |
| Yes | 434 (93.7) | 84 (91.3) |
| NR/ND | - | 2 (2.2) |
| **Goods transport/ construction** |  |  |
| No | 294 (63.5) | 34 (37.0) |
| Yes | 169 (36.5) | 56 (60.9) |
| NR/ND | - | 2 (2.2) |
| **People transport** |  |  |
| No | 287 (62.0) | 43 (46.7) |
| Yes | 176 (38.0) | 47 (51.1) |
| NR/ND | - | 2 (2.2) |
| **Water collection** |  |  |
| No | 361 (78.0) | 44 (47.8) |
| Yes | 102 (22.0) | 46 (50.0) |
| NR/ND | - | 2 (2.2) |
| **Waste transport** |  |  |
| No | 366 (79.0) | 57 (62.0) |
| Yes | 97 (21.0) | 33 (35.9) |
| NR/ND | - | 2 (2.2) |
| **Firewood transport** |  |  |
| No | 245 (52.9) | 15 (16.3) |
| Yes | 218 (47.1) | 75 (81.5) |
| NR/ND | - | 2 (2.2) |
| **Not started work** |  |  |
| No | 439 (94.8) | 90 (97.8) |
| Yes | 24 (5.2) | - |
| NR/ND | - | 2 (2.2) |
| **Animal travel to Senegal** | |  |
| **Travel to Senegal to *Lumos* (weekly markets)** | | |
| **Travel to *Lumos*** |  |  |
| No | 422 (91.1) | 89 (96.7) |
| Yes | 36 (7.8) | - |
| NR/ND | 5 (1.1) | 3 (3.3) |
| **Travel frequency** |  | NA |
| No travel to *Lumos* | 422 (91.1) |  |
| Weekly | 21 (4.5) |  |
| Monthly | 4 (0.9) |  |
| Every 2 months | 1 (0.2) |  |
| No specific interval | 10 (2.2) |  |
| NR/ND | 5 (1.1) |  |
| **Travel to Senegal for other reasons** | | |
| **Travel for other reasons ^a^** |  |  |
| No | 354 (76.5) | 82 (89.1) |
| Yes | 104 (22.5) | 7 (7.6) |
| NR/ND | 5 (1.1) | 3 (3.3) |
| **Travel frequency** |  |  |
| No travel for other reasons | 355 (76.7) | 82 (89.1) |
| Weekly | 18 (3.9) | 1 (1.1) |
| Monthly | 6 (1.3) | 1 (1.1) |
| Every 2-3 months | 1 (0.2) | - |
| No specific interval | 76 (16.4) | 5 (5.4) |
| NR/ND | 7 (1.5) | 3 (3.3) |
| **Equid work level** | | |
| **Dry season (November to May)** | | |
| **Work status** |  |  |
| No work | 250 (54.0) | 18 (19.6) |
| Work (1-7 days/ wk) | 199 (43.0) | 69 (75.0) |
| NR/ND | 14 (3.0) | 5 (5.5) |
| **Work days/ week** |  |  |
| Median (IQR) | 0.0 (0.0-4.0) | 2.0 (0.5-4.0) |
| **Work hours/ day** |  |  |
| Median (IQR) | 0.0 (0.0-2.0) | 3.0 (1.0-4.0) |
| **Rainy season (June to October)** | | |
| **Work status** |  |  |
| No work | 27 (5.8) | 0 (0.0) |
| Work (1-7 days/ wk) | 423 (91.4) | 87 (94.6) |
| NR/ND | 13 (2.8) | 5 (5.5) |
| **Work days/ week** |  |  |
| Median (IQR) | 7.0 (7.0-7.0) | 7.0 (7.0-7.0) |
| **Work hours/ day** |  |  |
| Median (IQR) | 6.0 (4.0-7.0) | 5.0 (3.0-6.0) |
| **Equid harness & cart** | |  |
| **Harness material** |  |  |
| Rope | 457 (98.7) | 89 (96.7) |
| Other ^b^ | 4 (0.9) | - |
| No harness | 1 (0.2) | - |
| NR/ND | 1 (0.2) | 3 (3.3) |
| **Head restraint or harness maintenance** |  |  |
| Never mended/ Buy replacement if issue | 444 (95.9) | 86 (93.5) |
| Pay for services | 1 (0.2) | - |
| Owner mends | 9 (1.9) | 3 (3.3) |
| NR/ND | 9 (1.9) | 3 (3.3) |
| **Cart** |  |  |
| No | 43 (9.3) | 18 (19.6) |
| Yes | 415 (89.6) | 70 (76.1) |
| NR/ND | 5 (1.1) | 4 (4.3) |
| **Cart maintenance** |  |  |
| Pay for services | 395 (85.3) | 61 (66.3) |
| Owner mends | 12 (2.6) | 6 (6.5) |
| Never mended/ Buy replacement if issue | 7 (1.5) | 1 (1.1) |
| No cart owned | 43 (9.3) | 18 (19.6) |
| NR/ND | 6 (1.3) | 6 (6.5) |
| **Equid housing and restraint** | |  |
| **Day** | | |
| **Restraint** |  |  |
| Loose | 50 (10.8) | 16 (17.4) |
| Tethered | 405 (87.5) | 73 (79.3) |
| Loose and tethered | 2 (0.4) | 1 (1.1) |
| Fenced area | 4 (0.9) | - |
| NR/ND | 2 (0.4) | 2 (2.2) |
| **Tethering method** |  |  |
| Not tethered | 54 (11.7) | 16 (17.4) |
| Fetlock or lower forelimb | 143 (30.9) | 61 (66.3) |
| Lower hindlimb | 1 (0.2) | 1 (1.1) |
| Neck | 211 (45.6) | 9 (9.8) |
| Neck and fetlock or lower forelimb | 25 (5.4) | 3 (3.3) |
| Tethered by head collar | 27 (5.8) | - |
| NR/ND | 2 (0.4) | 2 (2.2) |
| **Housing/ shelter** |  |  |
| No | 147 (31.7) | 63 (68.5) |
| Yes | 314 (67.8) | 26 (28.3) |
| NR/ND | 2 (0.4) | 3 (3.3) |
| **Night** | | |
| **Restraint** |  |  |
| Loose | 10 (2.2) | 8 (8.7) |
| Tethered | 447 (96.5) | 81 (88.0) |
| Fenced area | 2 (0.4) | - |
| NR/ND | 4 (0.9) | 3 (3.3) |
| **Tethering method** |  |  |
| Not tethered | 12 (2.6) | 8 (8.7) |
| Fetlock or lower forelimb | 157 (33.9) | 67 (72.8) |
| Lower hindlimb | 1 (0.2) | - |
| Neck | 232 (50.1) | 11 (12.0) |
| Neck and fetlock or lower forelimb | 25 (5.4) | 3 (3.3) |
| Fetlock or lower forelimb and hindlimb | 1 (0.2) | - |
| Neck and hindlimbs tied together | 1 (0.2) | - |
| Tethered by head collar | 27 (5.8) | - |
| NR/ND | 7 (1.5) | 3 (3.3) |
| **Housing/ shelter** |  |  |
| No | 128 (27.6) | 65 (70.7) |
| Yes | 331 (71.5) | 25 (27.2) |
| NR/ND | 4 (0.9) | 2 (2.2) |
| **Equid water source** | | |
| **Dry season** | | |
| **Borehole at home** |  |  |
| No | 428 (92.4) | 79 (85.9) |
| Yes | 33 (7.1) | 8 (8.7) |
| NR/ND | 2 (0.4) | 5 (5.4) |
| **Borehole in village** |  |  |
| No | 163 (35.2) | 33 (35.9) |
| Yes | 298 (64.4) | 54 (58.7) |
| NR/ND | 2 (0.4) | 5 (5.4) |
| **Well** |  |  |
| No | 277 (59.8) | 60 (65.2) |
| Yes | 184 (39.7) | 27 (29.3) |
| NR/ND | 2 (0.4) | 5 (5.4) |
| **Pump** |  |  |
| No | 443 (95.7) | 74 (80.4) |
| Yes | 18 (3.9) | 13 (14.1) |
| NR/ND | 2 (0.4) | 5 (5.4) |
| **Tap** |  |  |
| No | 146 (31.5) | 30 (32.6) |
| Yes | 315 (68.0) | 57 (62.0) |
| NR/ND | 2 (0.4) | 5 (5.4) |
| **River** |  | NA |
| No | 460 (99.4) |  |
| Yes | 1 (0.2) |  |
| NR/ND | 2 (0.4) |  |
| **Rainy season** | | |
| **Borehole at home** |  |  |
| No | 424 (91.6) | 81 (88.0) |
| Yes | 37 (8.0) | 6 (6.5) |
| NR/ND | 2 (0.4) | 5 (5.4) |
| **Borehole in village** |  |  |
| No | 165 (35.6) | 32 (34.8) |
| Yes | 296 (63.9) | 55 (59.8) |
| NR/ND | 2 (0.4) | 5 (5.4) |
| **Well** |  |  |
| No | 280 (60.5) | 59 (64.1) |
| Yes | 181 (39.1) | 28 (30.4) |
| NR/ND | 2 (0.4) | 5 (5.4) |
| **Pump** |  |  |
| No | 441 (95.2) | 72 (78.3) |
| Yes | 20 (4.3) | 15 (16.3) |
| NR/ND | 2 (0.4) | 5 (5.4) |
| **Tap** |  |  |
| No | 145 (31.3) | 30 (32.6) |
| Yes | 316 (68.3) | 57 (62.0) |
| NR/ND | 2 (0.4) | 5 (5.4) |
| **Rain water** |  |  |
| No | 399 (86.2) | 81 (88.0) |
| Yes | 62 (13.4) | 6 (6.5) |
| NR/ND | 2 (0.4) | 5 (5.4) |
| **Equid diet management** | | |
| **Forage** |  |  |
| (Groundnut) hay | 450 (97.2) | 81 (88.0) |
| Hay and grass | 1 (0.2) | 1 (1.1) |
| No forage offered | 3 (0.6) | 7 (7.6) |
| NR/ND | 9 (1.9) | 3 (3.3) |
| **Grazing ^c^ or browsing ^d^ activity** |  |  |
| No | 309 (66.7) | 41 (44.6) |
| Grazing | 142 (30.7) | 42 (45.7) |
| Browsing | 1 (0.2) | 2 (2.2) |
| Grazing and browsing | 4 (0.9) | 2 (2.2) |
| NR/ND | 7 (1.5) | 5 (5.4) |
| **Grazing ^c^, hrs/ day** |  |  |
| Median (IQR) | 5.0 (3.0-8.0) | 5.5 (4.0-12.0) |
| **Grazing ^c^/ Browsing ^d^: Restraint** |  |  |
| Tethered | 86 (18.6) | 27 (29.3) |
| Loose | 61 (13.2) | 19 (20.7) |
| No grazing/ browsing | 308 (66.5) | 41 (44.6) |
| NR/ND | 8 (1.7) | 5 (5.4) |
| **Concentrates** |  |  |
| No | 111 (24.0) | 52 (56.5) |
| Yes | 350 (75.6) | 33 (35.9) |
| NR/ND | 2 (0.4) | 7 (7.6) |
| **Other equids owned (excluding study animal)** | | |
| **Other equids** |  |  |
| No | 43 (9.3) | 20 (21.7) |
| Yes | 420 (90.7) | 70 (76.1) |
| NR/ND | - | 2 (2.2) |
| **Other horses, *n*** |  |  |
| Median (IQR) | 2.0 (1.0-3.0) | 0.0 (0.0-1.0) |
| **Other donkeys, *n*** |  |  |
| Median (IQR) | 1.0 (0.0-2.0) | 2.0 (1.0-3.0) |
| **Equid ownership** | | |
| **Time, years** |  |  |
| Median (IQR) | 20.0 (10.0-30.0) | 15.0 (7.0-20.0) |
| **Occupants per household** | | |
| **Total occupants, *n*** |  |  |
| Median (IQR) | 17.0 (12.0-27.0) | 15.0 (10.8-25.3) |
| **Total adult occupants (≥18 years), *n*** |  |  |
| Median (IQR) | 8.0 (5.0-15.0) | 8.00 (5.8-15.0) |
| **Total child occupants (<18 years), *n*** |  |  |
| Median (IQR) | 8.0 (6.0-15.0) | 7.0 (5.0-11.0) |

IQR=Interquartile Range; NR/ND=No response/ No data.

^a^ “Other reasons” for study animal to travel to Senegal included, for general transport, ceremonies, farming activities, to conduct business, to visit family, water collection, and to visit hospital; ^b^ “Other” materials used for harness included, leather or chains (+/- rope); ^c^ Graze=feeding on grass, forage or other low growing vegetation; ^d^ Browse=opportunistic feeding, including feeding on leaves, shoots or other high growing vegetation.
