# Supplementary material for: Exploring Histoplasma species seroprevalence and risk factors for seropositivity in The Gambia’s working equid population: Baseline analysis of the Tackling Histoplasmosis project dataset
Source: Front Vet Sci. 2024 Sep 19;11:1444887. doi: 10.3389/fvets.2024.1444887 (PMC11446873; doi:10.3389/fvets.2024.1444887)
Supplement: Supplementary file 6 [file Table_S6.docx]

**S6 Table.** Univariable logistic regression analysis results, examining associations between demographic and clinical variables amongst horses (*N*=463) and donkeys (*N*=92) in The Gambia and *Histoplasma* spp. seropositivity based on Latex Agglutination Test (LAT) result.

|  | HORSES, *N*=463 | | | | | DONKEYS, *N*=92 | | | | | |
| --- | --- | --- | --- | --- | --- | --- | --- | --- | --- | --- | --- |
| Variable | **Frequency, *n* (%)** | ***Histoplasma* spp. seropositive, *n* (%), total *N*=370 ^a^** | ***Histoplasma* spp. seronegative, *n* (%), total *N*=92 ^a^** | **Odds Ratio (95% CI)** | ***p-*value** | **Frequency, *n* (%)** | ***Histoplasma* spp. seropositive, *n* (%), total *N*=43** | | ***Histoplasma* spp. seronegative, *n* (%), total *N*=49** | **Odds Ratio (95% CI)** | ***p-*value** |
| Demographic | | | | | | | | | | | |
| Sex |  |  |  |  |  |  |  | |  |  |  |
| Male | 259 (55.9) | 191 (73.7) | 68 (26.3) | 1.00 |  | 43 (46.7) | 18 (41.9) | | 25 (58.1) | 1.00 |  |
| Female | 204 (44.1) | 179 (88.2) | 24 (11.8) | 2.66 (1.60-4.41) | <0.001* | 49 (53.3) | 25 (51.0) | | 24 (49.0) | 1.45 (0.63-3.30) | 0.38 |
| Age, years ^b^ |  |  |  |  |  |  |  | |  |  |  |
| <2.5^H^/ <3.5^D^ | 44 (9.5) | 30 (68.2) | 14 (31.8) | 0.50 (0.25-0.99) | 0.045* | 5 (5.4) | 2 (40.0) | | 3 (60.0) | 0.76 (0.12-4.78) | 0.77 |
| 2.5-4.5^H^/ 3.5-5.5^D^ | 46 (9.9) | 38 (82.6) | 8 (17.4) | 1.10 (0.49-2.46) | 0.82 | 8 (8.7) | 4 (50.0) | | 4 (50.0) | 1.14 (0.27-4.86) | 0.86 |
| ≥4.5^H^/ ≥5.5^D^ | 373 (80.6) | 302 (81.2) | 70 (18.8) | 1.00 |  | 79 (85.9) | 37 (46.8) | | 42 (53.2) | 1.00 |  |
| Reproductive history (*N*=204 mares) | | | | | | **Reproductive history (*N*=49 jennies)** | | | | | |
| Currently in foal ^c^ |  |  |  |  |  |  |  | |  |  |  |
| No | 83 (40.7) | 74 (89.2) | 9 (10.8) | 1.00 |  | 26 (53.1) | 10 (38.5) | | 16 (61.5) | 1.00 |  |
| Yes | 28 (13.7) | 24 (85.7) | 4 (14.3) | 0.73 (0.21-2.58) | 0.63 | 10 (20.4) | 5 (50.0) | | 5 (50.0) | 1.60 (0.37-6.96) | 0.53 |
| NR/ND | 93 (45.6) | 81 (88.0) | 11 (12.0) | 0.90 (0.35-2.28) | 0.82 | 13 (26.5) | 10 (76.9) | | 3 (23.1) | 5.33 (1.18-24.21) | 0.03* |
| Previously in foal ^c^ |  |  |  |  |  |  |  | |  |  |  |
| No | 59 (28.9) | 49 (83.1) | 10 (16.9) | 1.00 |  | 20 (40.8) | 8 (40.0) | | 12 (60.0) | 1.00 |  |
| Yes | 50 (24.5) | 48 (96.0) | 2 (4.0) | 4.90 (1.02-23.53) | 0.047* | 16 (32.7) | 7 (43.8) | | 9 (56.3) | 1.17 (0.31-4.42) | 0.82 |
| NR/ND or currently in foal | 95 (46.6) | 82 (87.2) | 12 (12.8) | 1.40 (0.56-3.47) | 0.47 | 13 (26.5) | 10 (76.9) | | 3 (23.1) | 5.00 (1.04-24.03) | 0.045* |
| Clinical examination | | | | | | | | | | | |
| Behaviour |  |  |  |  |  |  |  | |  |  |  |
| BAR | 440 (95.0) | 348 (79.3) | 91 (20.7) | 1.00 |  | 88 (95.7) | 42 (47.7) | | 46 (52.3) | 1.00 |  |
| QAR | 18 (3.9) | 17 (94.4) | 1 (5.6) | 4.45 (0.58-33.85) | 0.15** | 2 (2.2) | 1 (50.0) | | 1 (50.0) | 1.10 (0.07-18.07) | 0.95 |
| Dull | - | - | - | - | - | 1 (1.1) | 0 (0.0) | | 1 (100.0) | 0.00 (0.00-) | 1.00 |
| NR/ND | 5 (1.1) | 5 (100.0) | 0 (0.0) | - | 1.00 | 1 (1.1) | 0 (0.0) | | 1 (100.0) | 0.00 (0.00-) | 1.00 |
| BCS, 0-5 |  |  |  |  |  |  |  | |  |  |  |
| Median (IQR) | 3.0 (2.0-3.0) | - | - | 0.96 (0.70-1.31) | 0.79 | 3.0 (3.0-3.0) | - | | - | 0.97 (0.35-2.66) | 0.95 |
| Mucous membrane examination | | | | | | | | | | | |
| Colour |  |  |  |  |  |  |  |  | |  |  |
| Pale pink | 443 (95.7) | 355 (80.3) | 87 (19.7) | 1.00 |  | 87 (94.6) | 42 (48.3) | 45 (51.7) | | 1.00 |  |
| White | 17 (3.7) | 12 (70.6) | 5 (29.4) | 0.59 (0.20-1.71) | 0.33 | 5 (5.4) | 1 (20.0) | 4 (80.0) | | 0.27 (0.03-2.49) | 0.25 |
| NR/ND | 3 (0.6) | 3 (100.0) | 0 (0.0) | - | 1.00 | - | - | - | | - | - |
| Hydration |  |  |  |  |  |  |  |  | |  |  |
| Moist | 393 (84.9) | 307 (78.3) | 85 (21.7) | 1.00 |  | 76 (82.6) | 36 (47.4) | 40 (52.6) | | 1.00 |  |
| Tacky | 63 (13.6) | 57 (90.5) | 6 (9.5) | 2.63 (1.10-6.31) | 0.03* | 15 (16.3) | 6 (40.0) | 9 (60.0) | | 0.74 (0.24-2.29) | 0.60 |
| NR/ND | 7 (1.5) | 6 (85.7) | 1 (14.3) | 1.66 (0.20-13.99) | 0.64 | 1 (1.1) | 1 (100.0) | 0 (0.0) | | - | 1.00 |
| CRT, seconds |  |  |  |  |  |  |  |  | |  |  |
| <2 | 422 (91.1) | 334 (79.3) | 87 (20.7) | 1.00 |  | 85 (92.4) | 38 (44.7) | 47 (55.3) | | 1.00 |  |
| 2 | 35 (7.6) | 30 (85.7) | 5 (14.3) | 1.56 (0.59-4.15) | 0.37 | 7 (7.6) | 5 (71.4) | 2 (28.6) | | 3.09 (0.57-16.84) | 0.19** |
| >2 | 2 (0.4) | 2 (100.0) | 0 (0.0) | - | 1.00 | - | - | - | | - | - |
| NR/ND | 4 (0.9) | 4 (100.0) | 0 (0.0) | - | 1.00 | - | - | - | | - | - |
| Ocular and peri-orbital examination | | | | | | | | | | | |
| Ocular discharge |  |  |  |  |  |  | | | | | |
| No | 319 (68.9) | 251 (78.7) | 68 (21.3) | 1.00 |  |  |  |  |  |  |  |
| Yes | 140 (30.2) | 115 (82.7) | 24 (17.3) | 1.30 (0.78-2.17) | 0.32 |  |  |  |  |  |  |
| NR/ND | 4 (0.9) | 4 (100.0) | 0 (0.0) | - | 1.00 |  |  |  |  |  |  |
| Ocular pathology |  |  |  |  |  |  |  | |  |  |  |
| No | 420 (90.7) | 332 (79.2) | 87 (20.8) | 1.00 |  | 91 (98.9) | 43 (47.3) | | 48 (52.7) | 1.00 |  |
| Yes | 40 (8.6) | 35 (87.5) | 5 (12.5) | 1.83 (0.70-4.82) | 0.22 | 1 (1.1) | 0 (0.0) | | 1 (100.0) | 0.00 (0.00-) | 1.00 |
| NR/ND | 3 (0.6) | 3 (100.0) | 0 (0.0) | - | 1.00 | - | - | | - | - | - |
| Peri-orbital pathology |  |  |  |  |  |  |  | |  |  |  |
| No | 415 (89.6) | 330 (79.5) | 85 (20.5) | 1.00 |  | 89 (96.7) | 42 (47.2) | | 47 (52.8) | 1.00 |  |
| Yes | 45 (9.7) | 37 (84.1) | 7 (15.9) | 1.36 (0.59-3.16) | 0.47 | 3 (3.3) | 1 (33.3) | | 2 (66.7) | 0.56 (0.05-6.40) | 0.64 |
| NR/ND | 3 (0.6) | 3 (100.0) | 0 (0.0) | - | 1.00 | - | - | | - | - | - |
| Lymph node palpation | | | | | | | | | | | |
| Submandibular |  |  |  |  |  |  | | | | | |
| Within normal limits | 390 (84.2) | 315 (81.0) | 74 (19.0) | 1.00 |  |  |  |  |  |  |  |
| Enlarged | 68 (14.7) | 50 (73.5) | 18 (26.5) | 0.65 (0.36-1.18) | 0.16** |  |  |  |  |  |  |
| Abscessated | 2 (0.4) | 2 (100.0) | 0 (0.0) | - | 1.00 |  |  |  |  |  |  |
| Unable to palpate | 0 (0.0) | - | - | - | - |  |  |  |  |  |  |
| NR/ND | 3 (0.6) | 3 (100.0) | 0 (0.0) | - | 1.00 |  |  |  |  |  |  |
| Retropharyngeal |  |  |  |  |  |  |  |  |  |  |  |
| Within normal limits | 449 (97.0) | 359 (80.1) | 89 (19.9) | 1.00 |  |  |  |  |  |  |  |
| Enlarged | 11 (2.4) | 8 (72.7) | 3 (27.3) | 0.66 (0.17-2.54) | 0.55 |  |  |  |  |  |  |
| Abscessated | 0 (0.0) | - | - | - | - |  |  |  |  |  |  |
| Unable to palpate | 0 (0.0) | - | - | - | - |  |  |  |  |  |  |
| NR/ND | 3 (0.6) | 3 (100.0) | 0 (0.0) | - | 1.00 |  |  |  |  |  |  |
| Prescapular |  |  |  |  |  |  |  |  |  |  |  |
| Within normal limits | 456 (98.5) | 365 (80.2) | 90 (19.8) | 1.00 |  |  |  |  |  |  |  |
| Enlarged | 4 (0.9) | 2 (50.0) | 2 (50.0) | 0.25 (0.03-1.77) | 0.16** |  |  |  |  |  |  |
| Abscessated | 0 (0.0) | - | - | - | - |  |  |  |  |  |  |
| Unable to palpate | 0 (0.0) | - | - | - | - |  |  |  |  |  |  |
| NR/ND | 3 (0.6) | 3 (100.0) | 0 (0.0) | - | 1.00 |  |  |  |  |  |  |
| Popliteal |  |  |  |  |  |  |  |  |  |  |  |
| Within normal limits | 399 (86.2) | 316 (79.4) | 82 (20.6) | 1.00 |  |  |  |  |  |  |  |
| Enlarged | 1 (0.2) | 0 (0.0) | 1 (100.0) | 0.00 (0.00-) | 1.00 |  |  |  |  |  |  |
| Abscessated | 0 (0.0) | - | - | - | - |  |  |  |  |  |  |
| Unable to palpate | 5 (1.1) | 5 (100.0) | 0 (0.0) | - | 1.00 |  |  |  |  |  |  |
| NR/ND | 58 (12.5) | 49 (84.5) | 9 (15.5) | 1.41 (0.67-2.99) | 0.37 |  |  |  |  |  |  |
| Respiratory examination | | | | | | | | | | | |
| Nasal discharge |  |  |  |  |  |  |  | |  |  |  |
| No | 283 (61.1) | 234 (82.7) | 49 (17.3) | 1.00 |  | 76 (82.6) | 37 (48.7) | | 39 (51.3) | 1.00 |  |
| Yes | 176 (38.0) | 132 (75.4) | 43 (24.6) | 0.64 (0.41-1.02) | 0.06** | 16 (17.4) | 6 (37.5) | | 10 (62.5) | 0.63 (0.21-1.91) | 0.42 |
| NR/ND | 4 (0.9) | 4 (100.0) | 0 (0.0) | - | 1.00 | - | - | | - | - | - |
| Increased or abnormal tracheal sounds |  |  |  |  |  |  |  | |  |  |  |
| No | 344 (74.3) | 281 (81.9) | 62 (18.1) | 1.00 |  | 63 (68.5) | 31 (49.2) | | 32 (50.8) | 1.00 |  |
| Increased normal | 114 (24.6) | 84 (73.7) | 30 (26.3) | 0.62 (0.38-1.02) | 0.06** | 29 (31.5) | 12 (41.4) | | 17 (58.6) | 0.73 (0.30-1.77) | 0.49 |
| Decreased normal | 1 (0.2) | 1 (100.0) | 0 (0.0) | - | 1.00 | - | - | | - | - | - |
| Wheeze | 1 (0.2) | 1 (100.0) | 0 (0.0) | - | 1.00 | - | - | | - | - | - |
| NR/ND | 3 (0.6) | 3 (100.0) | 0 (0.0) | - | 1.00 | - | - | | - | - | - |
| Increased or abnormal thoracic sounds |  |  |  |  |  |  |  | |  |  |  |
| No | 375 (81.0) | 293 (78.1) | 82 (21.9) | 1.00 |  | 72 (78.3) | 33 (45.8) | | 39 (54.2) | 1.00 |  |
| Increased normal | 62 (13.4) | 53 (86.9) | 8 (13.1) | 1.85 (0.85-4.06) | 0.12** | 9 (9.8) | 4 (44.4) | | 5 (55.6) | 0.95 (0.24-3.81) | 0.94 |
| Wheeze | 4 (0.9) | 3 (75.0) | 1 (25.0) | 0.84 (0.09-8.18) | 0.88 | 1 (1.1) | 1 (100.0) | | 0 (0.0) | - | 1.00 |
| Crackles | 1 (0.2 | 1 (100.0) | 0 (0.0) | - | 1.00 | - | - | | - | - | - |
| NR/ND | 21 (4.5) | 20 (95.2) | 1 (4.8) | 5.60 (0.74-42.3) | 0.10** | 10 (10.9) | 5 (50.0) | | 5 (50.0) | 1.18 (0.32-4.44) | 0.81 |
| Musculoskeletal examination and hoof management | | | | | | | | | | | |
| Lameness |  |  |  |  |  |  |  | |  |  |  |
| No | 413 (89.2) | 329 (79.9) | 83 (20.1) | 1.00 |  | 83 (90.2) | 39 (47.0) | | 44 (53.0) | 1.00 |  |
| Yes | 38 (8.2) | 30 (78.9) | 8 (21.1) | 0.95 (0.42-2.14) | 0.89 | 8 (8.7) | 4 (50.0) | | 4 (50.0) | 1.13 (0.26-4.82) | 0.87 |
| NR/ND | 12 (2.5) | 11 (91.7) | 1 (8.3) | 2.78 (0.35-21.80) | 0.33 | 1 (1.1) | 0 (0.0) | | 1 (100.0) | 0.00 (0.00-) | 1.00 |
| Limb swelling |  |  |  |  |  |  |  | |  |  |  |
| No | 423 (91.4) | 339 (80.3) | 83 (19.7) | 1.00 |  | 423 (91.4) | 339 (80.3) | | 83 (19.7) | 1.00 |  |
| Yes | 34 (7.3) | 25 (73.5) | 9 (26.5) | 0.68 (0.31-1.51) | 0.34 | 34 (7.3) | 25 (73.5) | | 9 (26.5) | 0.68 (0.31-1.51) | 0.34 |
| NR/ND | 6 (1.3) | 6 (100.0) | 0 (0.0) | - | 1.00 | 6 (1.3) | 6 (100.0) | | 0 (0.0) | - | 1.00 |
| Abnormal hoof conformation |  |  |  |  |  |  |  | |  |  |  |
| No | 143 (30.9) | 111 (77.6) | 32 (22.4) | 1.00 |  | 32 (34.8) | 15 (46.9) | | 17 (53.1) | 1.00 |  |
| Yes | 317 (68.5) | 256 (81.0) | 60 (19.0) | 1.23 (0.76-2.00) | 0.40 | 60 (65.2) | 28 (46.7) | | 32 (53.3) | 0.99 (0.42-2.34) | 0.99 |
| NR/ND | 3 (0.6) | 3 (100.0) | 0 (0.0) | - | 1.00 | - | - | | - | - | - |
| Hoof maintenance ^d^ |  |  |  |  |  |  |  | |  |  |  |
| No | 33 (7.1) | 28 (84.8) | 5 (15.2) | 1.00 |  | 4 (4.3) | 3 (75.0) | | 1 (25.0) | 1.00 |  |
| Yes | 430 (92.9) | 342 (79.7) | 87 (20.3) | 0.70 (0.26-1.87) | 0.48 | 86 (93.5) | 1 (25.0) | | 46 (53.5) | 0.29 (0.03-2.90) | 0.29 |
| NR/ND | - | - | - | - | - | 2 (2.2) | 0 (0.0) | | 2 (100.0) | 0.00 (0.00-) | 1.00 |
| Owner trims hooves |  |  |  |  |  |  |  | |  |  |  |
| No | 409 (88.3) | 325 (79.7) | 83 (20.3) | 1.00 |  | 64 (69.6) | 30 (46.9) | | 34 (53.1) | 1.00 |  |
| Yes | 52 (11.2) | 43 (82.7) | 9 (17.3) | 1.22 (0.57-2.60) | 0.61 | 23 (25.0) | 12 (52.2) | | 11 (47.8) | 0.30 (0.03-2.96) | 0.30 |
| NR/ND | 2 (0.4) | 2 (100.0) | 0 (0.0) | - | 1.00 | 5 (5.4) | 1 (20.0) | | 4 (80.0) | 0.08 (0.00-1.95) | 0.12* |
| Farrier ^e^ trims hooves |  |  |  |  |  |  |  | |  |  |  |
| No | 323 (69.8) | 260 (80.7) | 62 (19.3) | 1.00 |  | 79 (85.9) | 38 (48.1) | | 41 (51.9) | 1.00 |  |
| Yes | 138 (29.8) | 108 (78.3) | 30 (21.7) | 0.86 (0.53-1.40) | 0.54 | 8 (8.7) | 4 (50.0) | | 4 (50.0) | 1.24 (0.48-3.21) | 0.66 |
| NR/ND | 2 (0.4) | 2 (100.0) | 0 (0.0) | - | 1.00 | 5 (5.4) | 1 (20.0) | | 4 (80.0) | 0.28 (0.03-2.68) | 0.27 |
| Ectoparasite management | | | | | |  |  | |  |  |  |
| Types |  |  |  |  |  |  |  | |  |  |  |
| No ectoparasites | 84 (18.1) | 66 (78.6) | 18 (21.4) | 1.00 |  | 72 (78.3) | 37 (51.4) | | 35 (48.6) | 1.00 |  |
| Ticks | 343 (74.1) | 274 (80.1) | 68 (19.9) | 1.10 (0.61-1.97) | 0.75 | 19 (20.7) | 6 (31.6) | | 13 (68.4) | 0.44 (0.15-1.28) | 0.13** |
| Flies | 5 (1.1) | 3 (60.0) | 2 (40.0) | 0.41 (0.06-2.64) | 0.35 | 1 (1.1) | 0 (0.0) | | 1 (100.0) | 0.00 (0.00-) | 1.00 |
| Flies and ticks | 26 (5.6) | 22 (84.6) | 4 (15.4) | 1.50 (0.46-4.91) | 0.50 | - | - | | - | - | - |
| NR/ND | 5 (1.1) | 5 (100.0) | 0 (0.0) | - | 1.00 | - | - | | - | - | - |
| Treatment |  |  |  |  |  |  |  | |  |  |  |
| No | 240 (51.8) | 192 (80.0) | 48 (20.0) | 1.00 |  | 60 (65.2) | 25 (41.7) | | 35 (58.3) | 1.00 |  |
| Yes | 207 (44.7) | 165 (80.1) | 41 (19.9) | 1.01 (0.63-1.60) | 0.98 | 30 (32.6) | 17 (56.7) | | 13 (43.3) | 1.83 (0.76-4.44) | 0.18** |
| NR/ND | 16 (3.5) | 13 (81.3) | 3 (18.8) | 1.08 (0.30-3.95) | 0.90 | 2 (2.2) | 1 (50.0) | | 1 (50.0) | 1.40 (0.08-23.46) | 0.82 |
| Endoparasite management | | | | | |  |  | |  |  |  |
| Treatment |  |  |  |  |  |  |  | |  |  |  |
| No | 70 (15.1) | 55 (78.6) | 15 (21.4) | 1.00 |  | 28 (30.4) | 14 (50.0) | | 14 (50.0) | 1.00 |  |
| Yes | 393 (84.9) | 315 (80.4) | 77 (19.6) | 1.12 (0.60-2.08) | 0.73 | 62 (67.4) | 29 (46.8) | | 33 (53.2) | 0.88 (0.36-2.15) | 0.78 |
| NR/ND | - | - | - | - | - | 2 (2.2) | 0 (0.0) | | 2 (100.0) | 0.00 (0.00-) | 1.00 |
| Anthelmintic drugs from vet pharmacy |  |  |  |  |  |  |  | |  |  |  |
| No | 186 (40.2) | 149 (80.5) | 36 (19.5) | 1.00 |  | 40 (43.5) | 18 (45.0) | | 22 (55.0) | 1.00 |  |
| Yes | 277 (59.8) | 221 (79.8) | 56 (20.2) | 0.95 (0.60-1.52) | 0.84 | 50 (54.3) | 25 (50.0) | | 25 (50.0) | 1.22 (0.53-2.81) | 0.64 |
| NR/ND | - | - | - | - | - | 2 (2.2) | 0 (0.0) | | 2 (100.0) | 0.00 (0.00-) | 1.00 |
| Anthelmintic drugs from other sources |  |  |  |  |  |  |  | |  |  |  |
| No | 246 (53.1) | 207 (84.1) | 39 (15.9) | 1.00 |  | 59 (64.1) | 28 (47.5) | | 31 (52.5) | 1.00 |  |
| Yes | 217 (46.9) | 163 (75.5) | 53 (24.5) | 0.58 (0.37-0.92) | 0.02* | 31 (33.7) | 15 (48.4) | | 16 (51.6) | 1.04 (0.44-2.48) | 0.93 |
| NR/ND | - | - | - | - | - | 2 (2.2) | 0 (0.0) | | 2 (100.0) | 0.00 (0.00-) | 1.00 |
| Anthelmintic drugs from other sources: Specify |  |  |  |  |  |  |  | |  |  |  |
| LA | 177 (38.2) | 134 (76.1) | 42 (23.9) | 1.00 |  | 23 (25.0) | 11 (47.8) | | 12 (52.2) | 1.00 |  |
| “Lumo”/ local trader | 21 (4.5) | 12 (57.1) | 9 (42.9) | 0.42 (0.17-1.06) | 0.07** | 4 (4.3) | 2 (50.0) | | 2 (50.0) | 1.09 (0.13-9.12) | 0.94 |
| LA and local trader | 6 (1.3) | 6 (100.0) | 0 (0.0) | - | 1.00 | - | - | | - | - | - |
| LA and GHDT | 1 (0.2) | 1 (100.0) | 0 (0.0) | - | 1.00 | - | - | | - | - | - |
| Don’t administer from other sources | 246 (53.1) | 207 (84.1) | 39 (15.9) | 1.66 (1.02-2.71) | 0.04* | 59 (64.1) | 28 (47.5) | | 31 (52.5) | 0.99 (0.38-2.59) | 0.98 |
| NR/ND | 12 (2.6) | 10 (83.3) | 2 (16.7) | 1.57 (0.33-7.44) | 0.57 | 6 (6.5) | 2 (33.3) | | 4 (66.7) | 0.55 (0.08-3.59) | 0.53 |
| Wounds (not Epizootic Lymphangitis) | | | | | | | | | | | |
| Wounds |  |  |  |  |  |  |  | |  |  |  |
| No | 357 (77.1) | 286 (80.1) | 71 (19.9) | 1.00 |  | 60 (65.2) | 32 (53.3) | | 28 (46.7) | 1.00 |  |
| Yes | 103 (22.2) | 81 (79.4) | 21 (20.6) | 0.96 (0.56-1.65) | 0.88 | 32 (34.8) | 11 (34.4) | | 21 (65.6) | 0.46 (0.19-1.11) | 0.09** |
| NR/ND | 3 (0.6) | 3 (100.0) | 0 (0.0) | - | 1.00 | - | - | | - | - | - |
| Wound treatment |  |  |  |  |  |  |  | |  |  |  |
| No wounds | 357 (77.1) | 286 (80.1) | 71 (19.9) | 1.00 |  | 60 (65.2) | 32 (53.3) | | 28 (46.7) | 1.00 |  |
| No | 59 (12.7) | 47 (81.0) | 11 (19.0) | 1.06 (0.52-2.15) | 0.87 | 26 (28.3) | 9 (34.6) | | 17 (65.4) | 0.46 (0.18-1.20) | 0.11** |
| Yes | 39 (8.4) | 30 (76.9) | 9 (23.1) | 0.83 (0.38-1.82) | 0.64 | 6 (6.5) | 2 (33.3) | | 4 (66.7) | 0.44 (0.07-2.57) | 0.36 |
| NR/ND | 8 (1.7) | 7 (87.5) | 1 (12.5) | 1.74 (0.21-14.35) | 0.61 | - | - | | - | - | - |
| Epizootic Lymphangitis history | | | | | | | | | | | |
| Previous EL ^f^ |  |  |  |  |  |  |  | |  |  |  |
| No | 324 (70.0) | 251 (77.7) | 72 (22.3) | 1.00 |  | 76 (82.6) | 35 (46.1) | | 41 (53.9) | 1.00 |  |
| Yes | 138 (29.8) | 118 (85.5) | 20 (14.5) | 1.69 (0.99-2.91) | 0.06** | 14 (15.2) | 8 (57.1) | | 6 (42.9) | 1.56 (0.49-4.94) | 0.45 |
| NR/ND | 1 (0.2) | 1 (100.0) | 0 (0.0) | - | 1.00 | 2 (2.2) | 0 (0.0) | | 2 (100.0) | 0.00 (0.00-) | 1.00 |
| Current EL ^f^ |  |  |  |  |  |  |  | | | | |
| No | 458 (98.9) |  |  |  |  | 89 (96.7) |  |  |  |  |  |
| Yes | 4 (0.9) |  |  |  |  | 1 (1.1) |  |  |  |  |  |
| NR/ND | 1 (0.2) |  |  |  |  | 2 (2.2) |  |  |  |  |  |

^H^=Horse, ^D^=Donkey; BAR=Bright alert responsive; QAR=Quiet alert responsive; BCS=Body Condition Score; CRT=Capillary Refill Time; EL=Epizootic Lymphangitis; LA=Livestock Agent; GHDT=Gambia Horse and Donkey Trust; IQR=Interquartile Range; NR/ND=No response/ No data; * *p*-value <0.50; ** *p*-value <0.20.

^a^ *n*=1 horse excluded based on missing serum sample (no LAT result); ^b^ Ageing based on incisor eruption times on dental examination; ^c^ Descriptive statistics and regression analyses performed for mares and jennies only (*N*=204); ^d^ “Hoof maintenance” includes the following: owner monitors foot and hoof shape, owner trims hooves, owner visits farrier to trim hooves, or other specified hoof management described by owner; ^e^ “Farriers” are defined as individuals with basic training in farriery (these are not the Livestock Agents and they may have other sources of incomes, such as farming); ^f^ Previous or current Epizootic Lymphangitis reported by owner in *any* or *other* owned equids (excluding the study animal), respectively.
