# Supplementary material for: Exploring Histoplasma species seroprevalence and risk factors for seropositivity in The Gambia’s working equid population: Baseline analysis of the Tackling Histoplasmosis project dataset
Source: Front Vet Sci. 2024 Sep 19;11:1444887. doi: 10.3389/fvets.2024.1444887 (PMC11446873; doi:10.3389/fvets.2024.1444887)
Supplement: Supplementary file 7 [file Table_S7.docx]

**S7 Table.** Univariable logistic regression analysis results, examining associations between haematological and biochemical parameters amongst horses (*N*=463) and donkeys (*N*=92) in The Gambia, and *Histoplasma* spp. seropositivity based on Latex Agglutination Test (LAT) result.

|  | | HORSES, *N*=463 | | DONKEYS, *N*=92 | | |
| --- | --- | --- | --- | --- | --- | --- |
| Variable | **Median (IQR)** | **Odds Ratio (95% CI)** | ***p-*value** | **Median (IQR)** | **Odds Ratio (95% CI)** | ***p-*value** |
| Packed Cell Volume, % | 30.0 (25.0-34.0) | 0.99 (0.95-1.02) | 0.49 | 32.0 (29.0-36.0) | 1.09 (1.01-1.19) | 0.04* |
| Total Protein, g/L | 78.0 (72.0-84.0) | 1.01 (0.98-1.03) | 0.57 | 80.0 (71.0-82.0) | 1.00 (0.95-1.06) | 0.88 |
| Neutrophil, % | 42.0 (20.0-54.9) | 0.99 (0.98-1.00) | 0.07** | 22.5 (16.8-32.7) | 1.00 (0.96-1.03) | 0.82 |
| Eosinophil, % | 5.0 (3.5-7.5) | 1.02 (0.95-1.10) | 0.58 | 6.0 (4.5-8.0) | 0.96 (0.83-1.11) | 0.57 |
| Basophil, % | 1.0 (0.5-2.0) | 1.14 (0.98-1.32) | 0.09** | 1.0 (0.5-2.3) | 0.88 (0.71-1.09) | 0.25 |
| Monocyte, % | 0.5 (0.0-1.3) | 0.97 (0.86-1.10) | 0.67 | 0.0 (0.0-1.5) | 0.89 (0.68-1.16) | 0.38 |
| Lymphocyte, % | 50.0 (37.5-68.9) | 1.01 (1.00-1.02) | 0.10** | 67.4 (53.6-75.0) | 1.01 (1.00-1.04) | 0.53 |

IQR=Interquartile Range; * *p*-value <0.50; ** *p*-value <0.20.

^a^ *n*=1 horse excluded based on missing serum sample (no LAT result).
