# Supplementary material for: Exploring Histoplasma species seroprevalence and risk factors for seropositivity in The Gambia’s working equid population: Baseline analysis of the Tackling Histoplasmosis project dataset
Source: Front Vet Sci. 2024 Sep 19;11:1444887. doi: 10.3389/fvets.2024.1444887 (PMC11446873; doi:10.3389/fvets.2024.1444887)
Supplement: Supplementary file 8 [file Table_S8.docx]

**S8 Table.** Univariable logistic regression analysis results, examining associations between geographic and seasonal variables amongst horses (*N*=463) and donkeys (*N*=92) in The Gambia and *Histoplasma* spp. seropositivity based on Latex Agglutination Test (LAT) result.

|  | | HORSES, *N*=463 | | | | DONKEYS, *N*=92 | | | | |
| --- | --- | --- | --- | --- | --- | --- | --- | --- | --- | --- |
| Variable | **Frequency, *n* (%)** | ***Histoplasma* spp. seropositive, *n* (%), total *N*=370 ^a^** | ***Histoplasma* spp. seronegative, n (%), total *N*=92 ^a^** | **Odds Ratio (95% CI)** | ***p-*value** | **Frequency, *n* (%)** | ***Histoplasma* spp. seropositive, *n* (%), total *N*=43** | ***Histoplasma* spp. seronegative, *n* (%), total *N*=49** | **Odds Ratio (95% CI)** | ***p-*value** |
| Geoclimatic | | | | | |  |  | | | |
| Region |  |  |  |  |  |  |  |  |  |  |
| URR | 134 (28.9) | 113 (85.0) | 20 (15.0) | 1.00 |  | 22 (23.9) | 13 (59.1) | 9 (40.9) | 1.00 |  |
| CRR/N | 99 (21.4) | 72 (72.7) | 27 (27.3) | 0.47 (0.25-0.90) | 0.02* | 1 (1.1) | 0 (0.0) | 1 (100.0) | 0.00 (0.00-) | 1.00 |
| CRR/S | 68 (14.7) | 51 (75.0) | 17 (25.0) | 0.53 (0.26-1.10) | 0.09** | 19 (20.7) | 3 (15.8) | 16 (84.2) | 0.13 (0.03-0.58) | 0.01* |
| NBR | 89 (19.2) | 75 (84.3) | 14 (15.7) | 0.95 (0.45-1.99) | 0.89 | 32 (34.8) | 15 (46.9) | 17 (53.1) | 0.61 (0.20-1.83) | 0.38 |
| LRR | 60 (13.0) | 48 (80.0) | 12 (20.0) | 0.71 (0.32-1.56) | 0.39 | 1 (1.1) | 1 (100.0) | 9 (0.0) | - | 1.00 |
| WCR | 13 (2.8) | 11 (84.6) | 2 (15.4) | 0.97 (0.20-4.73) | 0.97 | 17 (18.5) | 11 (64.7) | 6 (35.3) | 1.27 (0.34-4.70) | 0.72 |
| Season |  |  |  |  |  |  |  |  |  |  |
| Dry (November- May) | 325 (70.2) | 252 (77.8) | 72 (22.2) | 1.00 |  | 40 (43.5) | 19 (47.5) | 21 (52.5) | 1.00 |  |
| Rainy (June- October) | 138 (29.8) | 118 (85.5) | 20 (14.5) | 1.69 (0.98-2.90) | 0.06** | 52 (56.5) | 24 (46.2) | 28 (53.8) | 0.95 (0.42-2.16) | 0.90 |

URR=Upper River Region; CRR/N=Central River Region/ North; CRR/S=Central River Region/ South; NBR=North Bank Region; LRR=Lower River Region; WCR=West Coast Region; * *p*-value <0.50; ** *p*-value <0.20.

^a^ *n*=1 horse excluded based on missing serum sample (no LAT result).
