# Supplementary material for: Exploring Histoplasma species seroprevalence and risk factors for seropositivity in The Gambia’s working equid population: Baseline analysis of the Tackling Histoplasmosis project dataset
Source: Front Vet Sci. 2024 Sep 19;11:1444887. doi: 10.3389/fvets.2024.1444887 (PMC11446873; doi:10.3389/fvets.2024.1444887)
Supplement: Supplementary file 9 [file Table_S9.docx]

**S9 Table.** Univariable logistic regression analysis results, examining associations between variables pertaining to equid management practices and the compound environment, amongst horses (*N*=463) and donkeys (*N*=92) in The Gambia and *Histoplasma* spp. seropositivity based on Latex Agglutination Test (LAT) result.

|  | HORSES, *N*=463 | | | | | DONKEYS, *N*=92 | | | | |
| --- | --- | --- | --- | --- | --- | --- | --- | --- | --- | --- |
| Variable | **Frequency, n (%)** | ***Histoplasma* spp. seropositive, n (%), total *N*=370 ^a^** | ***Histoplasma* spp. seronegative, n (%), total *N*=92 ^a^** | **Odds Ratio (95% CI)** | ***p-*value** | **Frequency, *n* (%)** | ***Histoplasma* spp. seropositive, *n* (%), total *N*=43** | ***Histoplasma* spp. seronegative, *n* (%), total *N*=49** | **Odds Ratio (95% CI)** | ***p-*value** |
| Equid role | | | | | |  |  | | | |
| Ploughing |  |  |  |  |  |  |  |  |  |  |
| No | 29 (6.3) | 20 (69.0) | 9 (31.0) | 1.00 |  | 6 (6.5) | 2 (33.3) | 4 (66.7) | 1.00 |  |
| Yes | 433 (93.7) | 350 (80.8) | 83 (19.2) | 1.90 (0.83-4.32) | 0.13** | 84 (91.3) | 41 (48.8) | 43 (51.2) | 1.91 (0.33-10.98) | 0.47 |
| NR/ND | - | - | - | - | - | 2 (2.2) | 0 (0.0) | 2 (100.0) | 0.00 (0.00-) | 1.00 |
| Goods transport/ construction |  |  |  |  |  |  |  |  |  |  |
| No | 294 (63.5) | 249 (85.0) | 44 (15.0) | 1.00 |  | 34 (37.0) | 21 (61.8) | 13 (38.2) | 1.00 |  |
| Yes | 169 (36.5) | 121 (71.6) | 48 (28.4) | 0.45 (0.28-0.71) | <0.001* | 56 (60.9) | 22 (39.3) | 34 (60.7) | 0.40 (0.17-0.96) | 0.04* |
| NR/ND | - | - | - | - | - | 2 (2.2) | 0 (0.0) | 2 (100.0) | 0.00 (0.00-) | 1.00 |
| People transport |  |  |  |  |  |  |  |  |  |  |
| No | 287 (62.0) | 244 (85.3) | 42 (14.7) | 1.00 |  | 43 (46.7) | 26 (60.5) | 17 (39.5) | 1.00 |  |
| Yes | 176 (38.0) | 126 (71.6) | 50 (28.4) | 0.43 (0.27-0.69) | <0.001* | 47 (51.1) | 17 (36.2) | 30 (63.8) | 0.37 (0.16-0.87) | 0.02* |
| NR/ND | - | - | - | - | - | 2 (2.2) | 0 (0.0) | 2 (100.0) | 0.00 (0.00-) | 1.00 |
| Water collection |  |  |  |  |  |  |  |  |  |  |
| No | 361 (78.0) | 293 (81.4) | 67 (18.6) | 1.00 |  | 44 (47.8) | 21 (47.7) | 23 (52.3) | 1.00 |  |
| Yes | 102 (22.0) | 77 (75.5) | 25 (24.5) | 0.70 (0.42-1.19) | 0.19** | 46 (50.0) | 22 (47.8) | 24 (52.2) | 1.00 (0.44-2.30) | 0.99 |
| NR/ND | - | - | - | - | - | 2 (2.2) | 0 (0.0) | 2 (100.0) | 0.00 (0.00-) | 1.00 |
| Waste transport |  |  |  |  |  |  |  |  |  |  |
| No | 366 (79.0) | 293 (80.3) | 72 (19.7) | 1.00 |  | 57 (62.0) | 26 (45.6) | 31 (54.4) | 1.00 |  |
| Yes | 97 (21.0) | 77 (79.4) | 20 (20.6) | 0.95 (0.54-1.65) | 0.85 | 33 (35.9) | 17 (51.5) | 16 (48.5) | 1.27 (0.54-2.99) | 0.59 |
| NR/ND | - | - | - | - | - | 2 (2.2) | 0 (0.0) | 2 (100.0) | 0.00 (0.00-) | 1.00 |
| Firewood transport |  |  |  |  |  |  |  |  |  |  |
| No | 245 (52.9) | 209 (85.7) | 35 (14.3) | 1.00 |  | 15 (16.3) | 10 (66.7) | 5 (33.3) | 1.00 |  |
| Yes | 218 (47.1) | 161 (73.9) | 57 (26.1) | 0.47 (0.30-0.76) | 0.002* | 75 (81.5) | 33 (44.0) | 42 (56.0) | 0.39 (0.12-1.26) | 0.12** |
| NR/ND | - | - | - | - | - | 2 (2.2) | 0 (0.0) | 2 (100.0) | 0.00 (0.00-) | 1.00 |
| Not started work |  |  |  |  |  |  |  |  |  |  |
| No | 439 (94.8) | 353 (80.6) | 85 (19.4) | 1.00 |  | 90 (97.8) | 43 (47.8) | 47 (52.2) | 1.00 |  |
| Yes | 24 (5.2) | 17 (70.8) | 7 (29.2) | 0.59 (0.24-1.46) | 0.25 | - | - | - | - | - |
| NR/ND | - | - | - | - | - | 2 (2.2) | 0 (0.0) | 2 (100.0) | 0.00 (0.00-) | 1.00 |
| Animal travel to Senegal | | | | | |  |  | | | |
| Travel to Senegal to *Lumos* (weekly markets) | | | | | | | | | | |
| Travel to *Lumos* |  |  |  |  |  |  |  |  |  |  |
| No | 422 (91.1) | 342 (81.2) | 79 (18.8) | 1.00 |  | 89 (96.7) | 42 (47.2) | 47 (52.8) | 1.00 |  |
| Yes | 36 (7.8) | 25 (69.4) | 11 (30.6) | 0.53 (0.25-1.11) | 0.09** | - | - | - | - | - |
| NR/ND | 5 (1.1) | 3 (60.0) | 2 (40.0) | 0.35 (0.06-2.11) | 0.25 | 3 (3.3) | 1 (3.3) | 2 (66.7) | 0.56 (0.05-6.40) | 0.64 |
| Travel frequency |  |  |  |  |  |  |  |  |  |  |
| No travel to *Lumos* | 422 (91.1) | 342 (81.2) | 79 (18.8) | 1.00 |  |  |  |  |  |  |
| Weekly | 21 (4.5) | 13 (61.9) | 8 (38.1) | 0.38 (0.15-0.94) | 0.04* |  |  |  |  |  |
| Monthly | 4 (0.9) | 3 (75.0) | 1 (25.0) | 0.69 (0.07-6.75) | 0.75 |  |  |  |  |  |
| Every 2 months | 1 (0.2) | 1 (100.0) | 0 (0.0) | - | 1.00 |  |  |  |  |  |
| No specific interval | 10 (2.2) | 8 (80.0) | 2 (20.0) | 0.92 (0.19-4.44) | 0.92 |  |  |  |  |  |
| NR/ND | 5 (1.1) | 3 (60.0) | 2 (40.0) | 0.35 (0.06-2.11) | 0.25 |  |  |  |  |  |
| Travel to Senegal for other reasons | | | | | | | | | | |
| Travel for other reasons ^b^ |  |  |  |  |  |  |  |  |  |  |
| No | 354 (76.5) | 291 (82.4) | 62 (17.6) | 1.00 |  | 82 (89.1) | 39 (47.6) | 43 (52.4) | 1.00 |  |
| Yes | 104 (22.5) | 76 (73.1) | 28 (26.9) | 0.58 (0.35-0.97) | 0.04* | 7 (7.6) | 3 (42.9) | 4 (57.1) | 0.83 (0.17-3.93) | 0.81 |
| NR/ND | 5 (1.1) | 3 (60.0) | 2 (40.0) | 0.32 (0.05-1.95) | 0.22 | 3 (3.3) | 1 (33.3) | 2 (66.7) | 0.55 (0.05-6.32) | 0.63 |
| Frequency |  |  |  |  |  |  |  |  |  |  |
| No travel for other reasons | 355 (76.7) | 292 (82.5) | 62 (17.5) | 1.00 |  | 82 (89.1) | 39 (47.6) | 43 (52.4) | 1.00 |  |
| Weekly | 18 (3.9) | 12 (66.7) | 6 (33.3) | 0.43 (0.15-1.18) | 0.10** | 1 (1.1) | 1 (100.0) | 0 (0.0) | - | 1.00 |
| Monthly | 6 (1.3) | 6 (100.0) | 0 (0.0) | - | 1.00 | 1 (1.1) | 0 (0.0) | 1 (100.0) | 0.00 (0.00-) | 1.00 |
| Every 2-3 months | 1 (0.2) | 0 (0.0) | 1 (100.0) | - | 1.00 | - | - | - | - | - |
| No specific interval | 76 (16.4) | 55 (72.4) | 21 (27.6) | 0.56 (0.31-0.99) | 0.045* | 5 (5.4) | 2 (40.0) | 3 (60.0) | 0.74 (0.12-4.63) | 0.74 |
| NR/ND | 7 (1.5) | 5 (71.4) | 2 (28.6) | 0.53 (0.10-2.80) | 0.46 | 3 (3.3) | 1 (33.3) | 2 (66.7) | 0.55 (0.05-6.32) | 0.63 |
| Equid work level | | | | | |  |  | | | |
| Dry season (November to May) | | | | | | | | | | |
| Work status |  |  |  |  |  |  |  |  |  |  |
| No work | 250 (54.0) | 215 (86.3) | 34 (13.7) | 1.00 |  | 18 (19.6) | 9 (50.0) | 9 (50.0) | 1.00 |  |
| Work (1-7 days/ wk) | 199 (43.0) | 144 (72.4) | 55 (27.6) | 0.41 (0.26-0.67) | <0.001* | 69 (75.0) | 32 (46.4) | 37 (53.6) | 0.87 (0.31-2.44) | 0.78 |
| NR/ND | 14 (3.0) | 11 (78.6) | 3 (21.4) | 0.58 (0.15-2.19) | 0.42 | 5 (5.4) | 2 (40.0) | 3 (60.0) | 0.67 (0.09-4.99) | 0.69 |
| Work days/ week |  |  |  |  |  |  |  |  |  |  |
| Median (IQR) | 0.0 (0.0-4.0) | - | - | 0.95 (0.87-1.03) | 0.20 | 2.0 (0.5-4.0) | - | - | 1.02 (0.86-1.20) | 0.83 |
| Work hours/ day |  |  |  |  |  |  |  |  |  |  |
| Median (IQR) | 0.0 (0.0-2.0) | - | - | 0.81 (0.73-0.91) | <0.001* | 3.0 (1.0-4.0) | - | - | 1.00 (0.82-1.23) | 0.98 |
| Rainy season (June to October) | | | | | | | | | | |
| Work status |  |  |  |  |  |  |  |  |  |  |
| No work | 27 (5.8) | 18 (66.7) | 9 (33.3) | 1.00 |  | 0 (0.0) | - | - |  |  |
| Work (1-7 days/ wk) | 423 (91.4) | 342 (81.0) | 80 (19.0) | 2.14 (0.93-4.93) | 0.08** | 87 (94.6) | 41 (47.1) | 46 (52.9) |  |  |
| NR/ND | 13 (2.8) | 10 (76.9) | 3 (23.1) | 1.67 (0.37-7.61) | 0.51 | 5 (5.4) | 2 (40.0) | 3 (60.0) |  |  |
|  |  |  |  |  |  |  |  |  |  |  |
| Work days/ week |  |  |  |  |  |  |  |  |  |  |
| Median (IQR) | 7.0 (7.0-7.0) | - | - | 1.10 (0.97-1.23) | 0.13** | 7.0 (7.0-7.0) | - | - | 0.96 (0.63-1.47) | 0.86 |
| Work hours/ day |  |  |  |  |  |  |  |  |  |  |
| Median (IQR) | 6.0 (4.0-7.0) | - | - | 1.08 (0.98-1.18) | 0.12** | 5.0 (3.0-6.0) | - | - | 1.17 (0.93-1.46) | 0.17** |
| Equid harness & cart | | | | | |  |  | | | |
| Harness material |  |  |  |  |  |  |  |  |  |  |
| Rope | 457 (98.7) | 366 (80.3) | 90 (19.7) | 1.00 |  | 89 (96.7) | 42 (47.2) | 47 (52.8) |  |  |
| Other ^c^ | 4 (0.9) | 3 (75.0) | 1 (25.0) | 0.74 (0.08-7.18) | 0.79 | - | - | - |  |  |
| No harness | 1 (0.2) | 0 (0.0) | 1 (100.0) | 0.00 (0.00-) | 1.00 | - | - | - |  |  |
| NR/ND | 1 (0.2) | 1 (100.0) | 0 (0.0) | - | 1.00 | 3 (3.3) | 1 (33.3) | 2 (66.7) |  |  |
| Harness maintenance |  |  |  |  |  |  |  |  |  |  |
| Never mended/ Buy replacement if issue | 444 (95.9) | 354 (79.9) | 89 (20.1) | 1.00 |  | 86 (93.5) | 40 (46.5) | 46 (53.5) | 1.00 |  |
| Pay for services | 1 (0.2) | 1 (100.0) | 0 (0.0) | - | 1.00 | - | - | - | - | **-** |
| Owner mends | 9 (1.9) | 8 (88.9) | 1 (11.1) | 2.01 (0.25-16.29) | 0.51 | 3 (3.3) | 2 (66.7) | 1 (33.3) | 2.30 (0.20-26.32) | 0.50 |
| NR/ND | 9 (1.9) | 7 (77.8) | 2 (22.2) | 0.88 (0.18-4.31) | 0.88 | 3 (3.3) | 1 (33.3) | 2 (66.7) | 0.58 (0.05-6.58) | 0.66 |
| Cart |  |  |  |  |  |  |  |  |  |  |
| No | 43 (9.3) | 35 (81.4) | 8 (18.6) | 1.00 |  | 43 (9.3) | 35 (81.4) | 8 (18.6) | 1.00 |  |
| Yes | 415 (89.6) | 330 (79.7) | 84 (20.3) | 0.90 (0.40-2.01) | 0.79 | 415 (89.6) | 330 (79.7) | 84 (20.3) | 0.90 (0.40-2.01) | 0.79 |
| NR/ND | 5 (1.1) | 5 (100.0) | 0 (0.0) | - | 1.00 | 5 (1.1) | 5 (100.0) | 0 (0.0) | - | 1.00 |
| Cart maintenance |  |  |  |  |  |  |  |  |  |  |
| Pay for services | 395 (85.3) | 316 (80.2) | 78 (19.8) | 1.00 |  | 61 (66.3) | 27 (44.3) | 34 (55.7) |  |  |
| Owner mends | 12 (2.6) | 8 (66.7) | 4 (33.3) | 0.49 (0.15-1.68) | 0.26 | 6 (6.5) | 1 (16.7) | 5 (83.3) |  |  |
| Never mended/ Buy replacement if issue | 7 (1.5) | 5 (71.4) | 2 (28.6) | 0.62 (0.12-3.24) | 0.57 | 1 (1.1) | 1 (100.0) | 0 (0.0) |  |  |
| No cart owned | 43 (9.3) | 35 (81.4) | 8 (18.6) | 1.08 (0.48-2.42) | 0.85 | 18 (19.6) | 11 (61.1) | 7 (38.9) |  |  |
| NR/ND | 6 (1.3) | 6 (100.0) | 0 (0.0) | - | 1.00 | 6 (6.5) | 3 (50.0) | 3 (50.0) |  |  |
| Equid housing and restraint | | | | | |  |  | | | |
| Day | | | | | | | | | | |
| Restraint |  |  |  |  |  |  |  |  |  |  |
| Tethered | 405 (87.5) | 317 (78.3) | 88 (21.7) | 1.00 |  | 73 (79.3) | 36 (49.3) | 37 (50.7) | 1.00 |  |
| Loose | 50 (10.8) | 46 (93.9) | 3 (6.1) | 4.26 (1.29-14.01) | 0.02* | 16 (17.4) | 7 (43.8) | 9 (56.3) | 0.80 (0.27-2.38) | 0.69 |
| Loose and tethered | 2 (0.4) | 1 (50.0) | 1 (50.0) | 0.28 (0.02-4.48) | 0.37 | 1 (1.1) | 0 (0.0) | 1 (100.0) | 0.00 (0.00-) | 1.00 |
| Fenced area | 4 (0.9) | 4 (100.0) | 0 (0.0) | - | 1.00 | - | - | - | - | - |
| NR/ND | 2 (0.4) | 2 (100.0) | 0 (0.0) | - | 1.00 | 2 (2.2) | 0 (0.0) | 2 (100.0) | 0.00 (0.00-) | 1.00 |
| Housing/ shelter |  |  |  |  |  |  |  |  |  |  |
| No | 147 (31.7) | 120 (82.2) | 26 (17.8) | 1.00 |  | 63 (68.5) | 30 (47.6) | 33 (52.4) | 1.00 |  |
| Yes | 314 (67.8) | 249 (79.3) | 65 (20.7) | 0.83 (0.50-1.37) | 0.47 | 26 (28.3) | 12 (46.2) | 14 (53.8) | 0.94 (0.38-2.36) | 0.90 |
| NR/ND | 2 (0.4) | 1 (50.0) | 1 (50.0) | 0.22 (0.01-3.58) | 0.29 | 3 (3.3) | 1 (33.3) | 2 (66.7) | 0.55 (0.05-6.38) | 0.63 |
| Night | | | | | | | | | | |
| Restraint |  |  |  |  |  |  |  |  |  |  |
| Tethered | 447 (96.5) | 357 (80.0) | 89 (20.0) | 1.00 |  | 81 (88.0) | 39 (48.1) | 42 (51.9) | 1.00 |  |
| Loose | 10 (2.2) | 9 (90.0) | 1 (10.0) | 2.24 (0.28-17.94) | 0.45 | 8 (8.7) | 4 (50.0) | 4 (50.0) | 1.08 (0.25-4.60) | 0.92 |
| Fenced area | 2 (0.4) | 2 (100.0) | 0 (0.0) | - | 1.00 | - | - | - | - | - |
| NR/ND | 4 (0.9) | 2 (50.0) | 2 (50.0) | 0.25 (0.04-1.79) | 0.17** | 3 (3.3) | 0 (0.0) | 3 (100.0) | 0.00 (0.00-) | 1.00 |
| Housing/ shelter |  |  |  |  |  |  |  |  |  |  |
| No | 128 (27.6) | 102 (79.7) | 26 (20.3) | 1.00 |  | 65 (70.7) | 32 (49.2) | 33 (50.8) | 1.00 |  |
| Yes | 331 (71.5) | 267 (80.9) | 63 (19.1) | 1.08 (0.65-1.80) | 0.77 | 25 (27.2) | 11 (44.0) | 14 (56.0) | 0.81 (0.32-2.05) | 0.66 |
| NR/ND | 4 (0.9) | 1 (25.0) | 3 (75.0) | 0.09 (0.01-0.85) | 0.04* | 2 (2.2) | 0 (0.0) | 2 (100.0) | 0.00 (0.00-) | 1.00 |
| Equid water source | | | | | |  |  | | | |
| Dry season | | | | | | | | | | |
| Borehole at home |  |  |  |  |  |  |  |  |  |  |
| No | 428 (92.4) | 340 (79.6) | 87 (20.4) | 1.00 |  | 79 (85.9) | 38 (48.1) | 41 (51.9) | 1.00 |  |
| Yes | 33 (7.1) | 28 (84.8) | 5 (15.2) | 1.43 (0.54-3.82) | 0.47 | 8 (8.7) | 4 (50.0) | 4 (50.0) | 1.08 (0.25-4.62) | 0.92 |
| NR/ND | 2 (0.4) | 2 (100.0) | 0 (0.0) | - | 1.00 | 5 (5.4) | 1 (20.0) | 4 (80.0) | 0.27 (0.03-2.52) | 0.25 |
| Borehole in village |  |  |  |  |  |  |  |  |  |  |
| No | 163 (35.2) | 130 (80.2) | 32 (19.8) | 1.00 |  | 33 (35.9) | 13 (39.4) | 20 (60.6) | 1.00 |  |
| Yes | 298 (64.4) | 238 (79.9) | 60 (20.1) | 0.98 (0.61-1.58) | 0.92 | 54 (58.7) | 29 (53.7) | 25 (46.3) | 1.79 (0.74-4.30) | 0.197** |
| NR/ND | 2 (0.4) | 2 (100.0) | 0 (0.0) | - | 1.00 | 5 (5.4) | 1 (20.0) | 4 (80.0) | 0.39 (0.04-3.84) | 0.42 |
| Well |  |  |  |  |  |  |  |  |  |  |
| No | 277 (59.8) | 225 (81.2) | 52 (18.8) | 1.00 |  | 60 (65.2) | 30 (50.0) | 30 (50.0) | 1.00 |  |
| Yes | 184 (39.7) | 143 (78.1) | 40 (21.9) | 0.83 (0.52-1.31) | 0.42 | 27 (29.3) | 12 (44.4) | 15 (55.6) | 0.80 (0.32-1.99) | 0.63 |
| NR/ND | 2 (0.4) | 2 (100.0) | 0 (0.0) | - | 1.00 | 5 (5.4) | 1 (20.0) | 4 (80.0) | 0.25 (0.03-2.37) | 0.23 |
| Pump |  |  |  |  |  |  |  |  |  |  |
| No | 443 (95.7) | 353 (79.9) | 89 (20.1) | 1.00 |  | 74 (80.4) | 34 (45.9) | 40 (54.1) | 1.00 |  |
| Yes | 18 (3.9) | 15 (83.3) | 3 (16.7) | 1.26 (0.36-4.45) | 0.72 | 13 (14.1) | 8 (61.5) | 5 (38.5) | 1.88 (0.56-6.30) | 0.30 |
| NR/ND | 2 (0.4) | 2 (100.0) | 0 (0.0) | - | 1.00 | 5 (5.4) | 1 (20.0) | 4 (80.0) | 0.29 (0.03-2.76) | 0.28 |
| Tap |  |  |  |  |  |  |  |  |  |  |
| No | 146 (31.5) | 115 (793) | 30 (20.7) | 1.00 |  | 30 (32.6) | 14 (46.7) | 16 (53.3) | 1.00 |  |
| Yes | 315 (68.0) | 253 (80.3) | 62 (19.7) | 1.07 (0.65-1.74) | 0.80 | 57 (62.0) | 28 (49.1) | 29 (50.9) | 1.10 (0.46-2.68) | 0.83 |
| NR/ND | 2 (0.4) | 2 (100.0) | 0 (0.0) | - | 1.00 | 5 (5.4) | 1 (20.0) | 4 (80.0) | 0.29 (0.03-2.89) | 0.29 |
| River |  |  |  |  |  |  |  |  |  |  |
| No | 460 (99.4) | 367 (80.0) | 92 (20.0) | 1.00 |  |  |  |  |  |  |
| Yes | 1 (0.2) | 1 (100.0) | 0 (0.0) | - | 1.00 |  |  |  |  |  |
| NR/ND | 2 (0.4) | 2 (100.0) | 0 (0.0) | - | 1.00 |  |  |  |  |  |
| Rainy season | | | | | | | | | | |
| Borehole at home |  |  |  |  |  |  |  |  |  |  |
| No | 424 (91.6) | 337 (79.7) | 86 (20.3) | 1.00 |  | 81 (88.0) | 39 (48.1) | 42 (51.9) | 1.00 |  |
| Yes | 37 (8.0) | 31 (83.8) | 6 (16.2) | 1.32 (0.53-3.26) | 0.55 | 6 (6.5) | 3 (50.0) | 3 (50.0) | 1.08 (0.21-5.66) | 0.93 |
| NR/ND | 2 (0.4) | 2 (100.0) | 0 (0.0) | - | 1.00 | 5 (5.4) | 1 (20.0) | 4 (80.0) | 0.27 (0.03-2.51) | 0.25 |
| Borehole in village |  |  |  |  |  |  |  |  |  |  |
| No | 165 (35.6) | 131 (79.9) | 33 (20.1) | 1.00 |  | 32 (34.8) | 13 (40.6) | 19 (59.4) | 1.00 |  |
| Yes | 296 (63.9) | 237 (80.1) | 59 (19.9) | 1.01 (0.63-1.63) | 0.96 | 55 (59.8) | 29 (52.7) | 26 (47.3) | 1.63 (0.68-3.94) | 0.28 |
| NR/ND | 2 (0.4) | 2 (100.0) | 0 (0.0) | - | 1.00 | 5 (5.4) | 1 (20.0) | 4 (80.0) | 0.37 (0.04-3.65) | 0.39 |
| Well |  |  |  |  |  |  |  |  |  |  |
| No | 280 (60.5) | 228 (81.4) | 52 (18.6) | 1.00 |  | 59 (64.1) | 29 (49.2) | 30 (50.8) | 1.00 |  |
| Yes | 181 (39.1) | 140 (77.8) | 40 (22.2) | 0.80 (0.50-1.27) | 0.34 | 28 (30.4) | 13 (46.4) | 15 (53.6) | 0.90 (0.36-2.21) | 0.81 |
| NR/ND | 2 (0.4) | 2 (100.0) | 0 (0.0) | - | 1.00 | 5 (5.4) | 1 (20.0) | 4 (80.0) | 0.26 (0.03-2.45) | 0.24 |
| Pump |  |  |  |  |  |  |  |  |  |  |
| No | 441 (95.2) | 351 (79.8) | 89 (20.2) | 1.00 |  | 72 (78.3) | 33 (45.8) | 39 (54.2) | 1.00 |  |
| Yes | 20 (4.3) | 17 (85.) | 3 (15.0) | 1.44 (0.41-5.01) | 0.57 | 15 (16.3) | 9 (60.0) | 6 (40.0) | 1.77 (0.57-5.50) | 0.32 |
| NR/ND | 2 (0.4) | 2 (100.0) | 0 (0.0) | - | 1.00 | 5 (5.4) | 1 (20.0) | 4 (80.0) | 0.30 (0.03-2.78) | 0.29 |
| Tap |  |  |  |  |  |  |  |  |  |  |
| No | 145 (31.3) | 114 (79.2) | 30 (20.8) | 1.00 |  | 30 (32.6) | 14 (46.7) | 16 (53.3) | 1.00 |  |
| Yes | 316 (68.3) | 254 (80.4) | 62 (19.6) | 1.08 (0.66-1.76) | 0.76 | 57 (62.0) | 28 (49.1) | 29 (50.9) | 1.10 (0.46-2.68) | 0.83 |
| NR/ND | 2 (0.4) | 2 (100.0) | 0 (0.0) | - | 1.00 | 5 (5.4) | 1 (20.0) | 4 (80.0) | 0.29 (0.03-2.87) | 0.29 |
| Rain water |  |  |  |  |  |  |  |  |  |  |
| No | 399 (86.2) | 314 (78.9) | 84 (21.1) | 1.00 |  | 81 (88.0) | 40 (49.4) | 41 (50.6) | 1.00 |  |
| Yes | 62 (13.4) | 54 (87.1) | 8 (12.9) | 1.81 (0.83-3.94) | 0.14** | 6 (6.5) | 2 (33.3) | 4 (66.7) | 0.51 (0.09-2.96) | 0.46 |
| NR/ND | 2 (0.4) | 2 (100.0) | 0 (0.0) | - | 1.00 | 5 (5.4) | 1 (20.0) | 4 (80.0) | 0.26 (0.03-2.39) | 0.23 |
| Equid diet management | | | | | | | | | | |
| Forage | | | | | | | | | | |
| Type |  |  |  |  |  |  |  |  |  |  |
| (Groundnut) hay | 450 (97.2) | 361 (80.4) | 88 (19.6) | 1.00 |  | 81 (88.0) | 39 (48.1) | 42 (51.9) | 1.00 |  |
| Hay and grass | 1 (0.2) | 1 (100.0) | 0 (0.0) | - | 1.00 | 1 (1.1) | 0 (0.0) | 1 (100.0) | 0.00 (0.00-) | 1.00 |
| No forage offered | 3 (0.6) | 2 (66.7) | 1 (33.3) | 0.49 (0.04-5.44) | 0.56 | 7 (7.6) | 4 (57.1) | 3 (42.9) | 1.44 (0.30-6.83) | 0.65 |
| NR/ND | 9 (1.9) | 6 (66.7) | 3 (33.3) | 0.49 (0.12-1.99) | 0.32 | 3 (3.3) | 0 (0.0) | 3 (100.0) | 0.00 (0.00-) | 1.00 |
| Grazing ^d^ or browsing ^e^ activity |  |  |  |  |  |  |  |  |  |  |
| No | 309 (66.7) | 236 (76.4) | 73 (23.6) | 1.00 |  | 41 (44.6) | 22 (53.7) | 19 (46.3) | 1.00 |  |
| Yes | 147 (31.7) | 129 (88.4) | 17 (11.6) | 2.35 (1.33-4.15) | 0.003* | 46 (50.0) | 20 (43.5) | 26 (56.5) | 0.66 (0.29-1.55) | 0.34 |
| NR/ND | 7 (1.5) | 5 (71.4) | 2 (28.6) | 0.77 (0.15-4.07) | 0.76 | 5 (5.4) | 1 (20.0) | 4 (80.0) | 0.22 (0.02-2.10) | 0.19** |
| Grazing ^d^, hrs/ day |  |  |  |  |  |  |  |  |  |  |
| Median (IQR) | 5.0 (3.0-8.0) | - | - | 0.98 (0.87-1.10) | 0.72 | 5.5 (4.0-12.0) | - | - | 1.02 (0.91-1.14) | 0.75 |
| Grazing ^d^/ Browsing ^e^: Restraint |  |  |  |  |  |  |  |  |  |  |
| No grazing/ browsing | 308 (66.5) | 235 (76.3) | 73 (23.7) | 1.00 |  | 41 (44.6) | 22 (53.7) | 19 (46.3) | 1.97 (0.73-5.32) | 0.18** |
| Tethered | 86 (18.6) | 75 (87.2) | 11 (12.8) | 2.12 (1.07-4.20) | 0.03* | 27 (29.3) | 10 (37.0) | 17 (63.0) | 1.00 |  |
| Loose | 61 (13.2) | 55 (91.7) | 5 (8.3) | 3.42 (1.32-8.86) | 0.01* | 19 (20.7) | 10 (52.6) | 9 (47.4) | 1.89 (0.57-6.22) | 0.30 |
| NR/ND | 8 (1.7) | 5 (62.5) | 3 (37.5) | 0.52 (0.12-2.22) | 0.38 | 5 (5.4) | 1 (20.0) | 4 (80.0) | 0.43 (0.04-4.35) | 0.47 |
| Concentrates | | | | | | | | | | |
| Concentrates |  |  |  |  |  |  |  |  |  |  |
| No | 111 (24.0) | 92 (82.9) | 19 (17.1) | 1.00 |  | 52 (56.5) | 29 (55.8) | 23 (44.2) | 1.00 |  |
| Yes | 350 (75.6) | 276 (79.1) | 73 (20.9) | 0.78 (0.45-1.36) | 0.38 | 33 (35.9) | 13 (39.4) | 20 (60.6) | 0.52 (0.21-1.25) | 0.14** |
| NR/ND | 2 (0.4) | 2 (100.0) | 0 (0.0) | - | 1.00 | 7 (7.6) | 1 (14.3) | 6 (85.7) | 0.13 (0.02-1.18) | 0.07** |
| Other equids owned (excluding study animal) | | | | | |  |  | | | |
| Other horses |  |  |  |  |  |  |  |  |  |  |
| No | 114 (24.6) | 90 (78.9) | 24 (21.1) | 1.00 |  | 65 (70.7) | 36 (55.4) | 29 (44.6) | 1.00 |  |
| Yes | 349 (75.4) | 280 (80.5) | 68 (19.5) | 1.10 (0.65-1.85) | 0.73 | 25 (27.2) | 7 (28.0) | 18 (72.0) | 0.31 (0.12-0.85) | 0.02* |
| NR/ND | - | - | - | - | - | 2 (2.2) | 0 (0.0) | 2 (100.0) | 0.00 (0.00-) | 1.00 |
| Other horses, *n* |  |  |  |  |  |  |  |  |  |  |
| Median (IQR) | 2.0 (1.0-3.0) | - | - | 0.95 (0.88-1.03) | 0.23 | 0.0 (0.0-1.0) | - | - | 0.56 (0.31-1.02) | 0.06** |
| Other donkeys |  |  |  |  |  |  |  |  |  |  |
| No | 162 (35.0) | 127 (78.4) | 35 (21.6) | 1.00 |  | 26 (28.3) | 10 (38.5) | 16 (61.5) | 1.00 |  |
| Yes | 300 (64.8) | 242 (80.9) | 57 (19.1) | 1.17 (0.73-1.88) | 0.52 | 64 (69.6) | 33 (51.6) | 31 (48.4) | 1.70 (0.67-4.32) | 0.26 |
| NR/ND | 1 (0.2) | 1 (100.0) | 0 (0.0) | - | 1.00 | 2 (2.2) | 0 (0.0) | 2 (100.0) | 0.00 (0.00-) | 1.00 |
| Other donkeys, *n* |  |  |  |  |  |  |  |  |  |  |
| Median (IQR) | 1.0 (0.0-2.0) | - | - | 1.10 (0.93-1.29) | 0.28 | 2.0 (1.0-3.0) | - | - | 1.11 (0.94-1.31) | 0.24 |
| Equid ownership | | | | | |  |  | | | |
| Time, years |  |  |  |  |  |  |  |  |  |  |
| Median (IQR) | 20.0 (10.0-30.0) | - | - | 1.01 (0.99-1.03) | 0.35 | 15.0 (7.0-20.0) | - | - | 1.00 (0.96-1.04) | 0.84 |
| Household | | | | | |  |  | | | |
| Total occupants, *n* |  |  |  |  |  |  |  |  |  |  |
| Median (IQR) | 17.0 (12.0-27.0) | - | - | 1.00 (0.99-1.02) | 0.77 | 15.0 (10.8-25.3) | - | - | 0.99 (0.96-1.03) | 0.58 |
| Total adult occupants (≥18 years), *n* |  |  |  |  |  |  |  |  |  |  |
| Median (IQR) | 8.0 (5.0-15.0) | - | - | 1.02 (0.99-1.05) | 0.27 | 8.00 (5.8-15.0) | - | - | 0.96 (0.91-1.02) | 0.21 |
| Total child occupants (<18 years), *n* |  |  |  |  |  |  |  |  |  |  |
| Median (IQR) | 8.0 (6.0-15.0) | - | - | 0.99 (0.96-1.02) | 0.48 | 7.0 (5.0-11.0) | - | - | 1.02 (0.95-1.09) | 0.66 |

IQR=Interquartile range; NR/ND=No response/ No data; * *p*-value <0.50; ** *p*-value <0.20.

^a^ *n*=1 horse excluded based on missing serum sample (no LAT result); ^b^ “Other reasons” for study animal to travel to Senegal included, for general transport, ceremonies, farming activities, conduct business, visit family, water collection, and visit hospital; ^c^ “Other” materials used for harness included, leather or chains (+/- rope); ^d^ Graze=feeding on grass, forage or other low growing vegetation; ^e^ Browse=opportunistic feeding, including feeding on leaves, shoots or other high growing vegetation.
