# Supplementary material for: Exploring Histoplasma species seroprevalence and risk factors for seropositivity in The Gambia’s working equid population: Baseline analysis of the Tackling Histoplasmosis project dataset
Source: Front Vet Sci. 2024 Sep 19;11:1444887. doi: 10.3389/fvets.2024.1444887 (PMC11446873; doi:10.3389/fvets.2024.1444887)
Supplement: Supplementary file 10 [file Table_S10.docx]

**S10 Table.** Multi-level logistic regression analysis examining variable associations with *Histoplasma* spp. seropositivity based on Latex Agglutination Test (LAT) results, amongst donkeys (*N*=92) in The Gambia. Household and study site are included as random effects.

| Variable | Frequency, *n* (%), total *N*=92 | *Histoplasma* spp. seropositive, *n* (%), *N*=43 | *Histoplasma* spp. seronegative, *n* (%), *N*=49 | Odds Ratio (95% CIs) | *p-*value |
| --- | --- | --- | --- | --- | --- |
| Main effects | | | | | |
| Horses owned |  |  |  |  |  |
| No | 65 (70.7) | 36 (55.4) | 29 (44.6) | 1.00 |  |
| Yes | 25 (27.2) | 7 (28.0) | 18 (72.0) | 0.23 (0.06-0.85) | 0.027* |
| NR/ND | 2 (2.2) | 0 (0.0) | 2 (100.0) | - | 1.00 |
| Random effects | | | | | |
| Household |  | | | | |
| Variance (Standard Error) | 0.00 (0.00) | | | | |
| Study site |  | | | | |
| Variance (Standard Error) | 0.65 (0.63) | | | | |

* *p*<0·05 (statistically significant).
